# Effect of Exposure to Gun Violence in Video Games on Children’s Dangerous Behavior With Real Guns: A Randomized Clinical Trial

**DOI:** 10.1001/jamanetworkopen.2019.4319

**Published:** 2019-05-31

**Authors:** Justin H. Chang, Brad J. Bushman

**Affiliations:** 1 School of Communication, The Ohio State University, Columbus; 2 Department of Psychology, The Ohio State University, Columbus

## Abstract

**Importance:**

Among resource-rich countries, the United States has the highest rate of child mortality by unintentional firearm use.

**Objective:**

To test whether children’s exposure to violent video games increases dangerous behavior around firearms.

**Design, Setting, and Participants:**

This randomized clinical trial was set in a university laboratory and included pairs of children aged 8 to 12 years who knew each other. Of 313 participants who signed up, 250 were tested (2 arrived without partners, 61 did not arrive to participate). Of the 250 children tested, 8 were excluded (2 did not complete the study, 2 had participated in a related study, and 4 were outliers). Each child was paid $25. Data were collected July 1, 2017, to July 31, 2018.

**Interventions:**

In a 3-group randomized design, pairs of children played or watched 1 of 3 versions of the game *Minecraft* for 20 minutes: (1) violent with guns, (2) violent with swords, or (3) nonviolent. The pairs of children were then placed in a different room and were told they could play with toys and games for 20 minutes. A cabinet in the room contained 2 hidden disabled handguns with counters for trigger pulls. Play sessions were videotaped.

**Main Outcomes and Measures:**

Main outcomes were touching a handgun, seconds spent holding a handgun, and number of trigger pulls (including at oneself or the partner). Control variables included sex, age, trait aggressiveness, exposure to violent media, attitudes toward guns, presence of firearms in the home, interest in firearms, and whether the child had taken a firearm safety course.

**Results:**

Of 242 participants, 220 children (mean [SD] age, 9.9 [1.4] years; 129 [58.6%] boys) found a gun and were included in analysis. Among the 76 children who played the video game that included gun violence, 47 children (61.8%) touched a handgun. Among the 74 children who played the video game that included sword violence, 42 (56.8%) touched a handgun. Among the 70 children who played the nonviolent video game, 31 (44.3%) touched a handgun. Participants who played a violent version of the game were more likely to shoot at themselves or their partners than those who played a nonviolent game. Other risk factors for dangerous behavior around firearms included self-reported habitual exposure to violent media and trait aggressiveness. Self-reported exposure to violent media was positively associated with total trigger pulls (incidence rate ratio [IRR], 1.40; 95% CI, 1.00-1.98) and trigger pulls at oneself or one’s partner (IRR, 1.88; 95% CI, 1.29-2.72). Trait aggression was positively associated with total trigger pulls (IRR, 13.52; 95% CI, 3.14-58.29), trigger pulls at oneself or one’s partner (IRR, 25.69; 95% CI, 5.92-111.39), and time spent holding a handgun (IRR, 4.22; 95% CI, 1.62-11.02). One protective factor was having taken a firearm safety training course.

**Conclusions and Relevance:**

Exposure to violent video games increases children’s dangerous behavior around firearms.

**Trial Registration:**

ClinicalTrials.gov identifier NCT03259139

## Key Points

**Question:**

Does exposure to violent video games cause children to engage in dangerous behavior around real firearms?

**Findings:**

In this randomized clinical trial, 220 children aged 8 to 12 years were assigned to play a video game in 1 of 3 conditions: with gun violence, with sword violence, or with no violence. Compared with children who played a game that was nonviolent, children who played a video game that included violence with guns or swords were more likely to touch a real, disabled handgun, handle a handgun longer, and pull the trigger more times, including at themselves or their partner. Reported habitual exposure to violent media was also a risk factor for dangerous behavior around firearms.

**Meaning:**

Exposure to violent video games increases children’s dangerous behavior around real firearms.

## Teaser

This randomized clinical trial explores whether children’s exposure to a violent video game increases dangerous behavior around firearms.

## Introduction

Each day in the United States, nearly 50 children and teenagers are shot with a firearm,^1^ often as a result of a child finding one loaded and unsecured.^2^ Among firearm-owning households with children, approximately 20% keep at least 1 firearm loaded and unsecured.^3^ Children in the United States are at least 10-fold more likely to be killed by an unintentional firearm discharge compared with children in other resource-rich countries.^4^

If children find an unsecured firearm at home, many factors can influence whether they will play with it. Our study focused on exposure to gun violence in media, in this case a video game, and controlled for several other potential factors. Based on social learning theory,^5^ we hypothesized that children exposed to gun violence in violent media would mimic that behavior.

According to social learning theory,^5^ people learn strategies for successful behaviors by direct experience and by observing others. According to this theory, people observe and copy or imitate the behavior of others, called *modeling*. The model can be a real person or a fictional character. Importantly for this study, modeling is especially likely to occur when the potential outcomes for a behavior are dangerous. Identification with the model is an important factor: the more a person identifies with the model, the more likely they are to internalize and emulate that behavior, especially if the model is rewarded for performing the behavior.^6^

In a 2017 randomized clinical trial conducted in our laboratory,^7^ 104 children aged 8 to 12 years were tested in pairs and randomly assigned to watch a 20-minute movie clip with or without guns. Next, children were placed in a different room with a hidden camera and were told that they could play with the toys and games in the room for 20 minutes. A cabinet in the room contained a real, disabled handgun. Children who watched the movie clip with guns held the handgun longer and pulled the trigger more times than those who saw the same movie without guns.^7^

This study seeks to replicate those findings using a violent video game and including an active participant (the player) and a passive participant (the watcher). Using a video game as a stimulus presents an interesting opportunity because participants can learn from their direct experience (playing the game) and from modeling (observing their avatar in the game). Based on a 2008 study,^8^ we predicted stronger effects for players than for watchers. To distinguish between gun violence and generic violence, we had 2 violent conditions—1 with guns and 1 with swords—and a nonviolent control condition. We hypothesized the most dangerous behaviors in the gun violence condition, then the sword violence condition, and then the nonviolent control condition.

## Methods

### Ethical Review

The study was approved by The Ohio State University Institutional Review Board. Parents or guardians were made aware of the full purpose of the experiment prior to giving written informed consent (rate = 100%) online via Qualtrics. All of the children provided assent in the laboratory via a signed form but were not informed of the full purpose of the experiment until the end. Participants and parents or guardians were informed that they could end the study at any time for any reason. The handguns were inspected by the campus police chief. This study followed Consolidated Standards of Reporting Trials (CONSORT) reporting guideline. The trial protocol is available in Supplement 1.

### Participants

Inclusion criteria included children aged 8 to 12 years. Children were tested in pairs and were either related or friends. Of these, 15 pairs were excluded (1 pair ended the study early, 1 pair had participated in the movie study,^7^ 11 pairs did not find the handguns, and 2 pairs were outliers), leaving a total of 220 participants (Figure).

**Figure.**
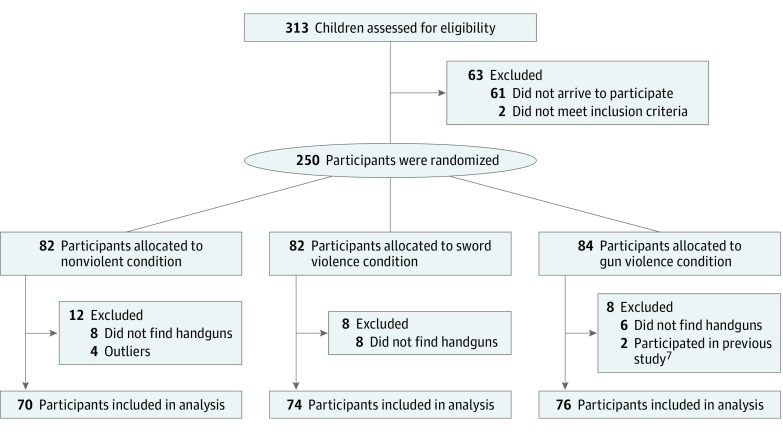
Flow Diagram of Participants

Participants were recruited through ResearchMatch (Vanderbilt University), Craigslist and Facebook ads, and referrals from other participants. One of us (J.H.C.) enrolled and scheduled participants. To be included, the participant had to bring another child aged 8 to 12 years (eg, sibling, cousin, friend). Each participant was paid $25.

### Procedure

Data were collected from July 1, 2017, to July 31, 2018. Participants were told the study was about what children like to do in their spare time, such as playing with video games, board games, and toys. Participants were tested in our laboratory at The Ohio State University. Participants filled out several self-report surveys while their parents or guardians provided demographic information and answered questions about guns.

In the self-report surveys, participants listed their 3 favorite video games, films, and television shows and how often they watched or played them, scored as 1 indicating rarely; 2, sometimes; or 3, often.^9^ We measured exposure to violent content as described in our 2017 movie study.^7^ We used Entertainment Software Rating Board^10^ ratings for video games, Motion Picture Association of America^11^ ratings for films, and Common Sense Media^12^ ratings for television shows. Each title was scored using a 7-point scale from 1 indicating nonviolent to 7, extremely violent. We multiplied the frequency of engagement by media rating and calculated the mean across media types (Cronbach α = .76).

Participants also completed a 9-item measure of how often they engaged in aggressive behaviors (eg, yelling, arguing, kicking, hitting), with each item scored as 0 indicating never; 1, sometimes; or 2, often (Cronbach α = .77).^13^ They also completed a 15-item measure of their attitudes toward guns (eg, “Carrying a gun makes people feel safe”), with each item scored from 0 indicating strongly disagree to 4, strongly agree (Cronbach α = .86).^14,15^

Using a random number generator, 1 of us (J.H.C) assigned pairs of participants to 1 of 3 versions of the video game *Minecraft* (Microsoft Corporation) on a computer for 20 minutes: (1) a violent version with guns that could be used to kill monsters, (2) a violent version with swords that could be used to kill monsters, or (3) a nonviolent version with no weapons and no monsters. The versions were otherwise identical. Participants played by themselves in a room with a hidden camera. One child within the pair was randomly assigned to play the game (active player) while the other watched (passive watcher). To avoid possible resentment, the child who watched the game was told they could play the game later if they wanted to. The player was instructed to find as many emeralds as possible, which are valuable objects hidden in chests within the game. *Minecraft* captures gameplay statistics, including the number of monsters killed and the number of chests opened. During this time, parents or guardians completed demographic questionnaires, estimated their child’s interest in firearms (0 indicating not at all interested to 4, very interested), reported how many firearms were in the home, and reported whether the child had taken a firearm safety course.

After gameplay, both participants reported if they had played or watched someone else play the game before (0 = no or 1 = yes); rated how familiar they were with the game; how exciting, boring, fun, and violent it was; how much they liked it; whether they felt like they were part of the action; and whether they wanted to play the game more (0 indicating not at all to 4, very much).

Next, participants went to a different room and were told they could play for 20 minutes with any toys (eg, Lego bricks, Nerf guns, foam swords) and games (eg, checkers, Jenga, UNO) in the room. The door of the room was closed while children were inside. A cabinet in the room contained 2 disabled 9-mm handguns with counters for trigger pulls (eAppendix in Supplement 2). Parents or guardians and 1 of us (J.H.C.) monitored the children in a separate room using a stationary hidden camera. A research assistant waited outside the door in case the children had questions. Finally, participants were thoroughly debriefed with the parent or guardian present. The debriefing included a discussion of firearm safety and information about counseling resources. The study lasted about 1 hour. One week later, the parents or guardians were contacted to ensure there were no adverse effects of participation, and none were reported. A more detailed description of the procedure is in the full study protocol in Supplement 1.

This study design has 4 advantages over the Dillon and Bushman^7^ design. First, the addition of a violent condition with swords allowed us to parse between gun violence and generic violence. Second, the conditions are all identical except for the presence or absence of weapons and monsters. Third, the inclusion of 2 firearms allowed both children to handle and pull the trigger at the same time, reducing variance that may be introduced by 1 child monopolizing time with a gun. Fourth, we tested whether taking a firearm safety course was associated with safer behavior around firearms.

### Coding

Three trained research assistants, blind to video game condition and experimental hypotheses, independently coded the play session videos. They coded whether the participants found the handguns. If the participants found the handguns, the research assistants coded whether the participants told an adult, whether the participants touched a handgun, how long the participants held the handgun, the number of trigger pulls, and the number trigger pulls while pointing a handgun at themselves or their partners. Random quality assurance checks and trigger pull counters confirmed codings.

### Statistical Analysis

Negative binomial regression models were used for the multivariate analysis. The negative binomial model, like the Poisson regression model, is appropriate for modeling-dependent measures that are counts. However, negative binomial regression is better suited for overdispersed data (ie, the dependent measure has an SD greater than its mean), whereas Poisson regression requires the mean and SD to be equal.

The primary outcome measures were whether a child touched a handgun, seconds spent holding a handgun, total trigger pulls, and trigger pulls while pointing a handgun at oneself or another. We controlled for several factors that can influence dangerous behavior around guns, including sex, age, trait aggressiveness, violent media consumption, whether the participant had taken a firearm safety course, firearms in the home, attitudes toward guns, and interest in firearms. The independent variable in all models was the video game version. As in our previous study,^7^ we tested 2 sets of models: (1) a reduced model that included only video game version and participant’s sex and (2) a full model that included video game version and participant covariates (ie, sex, age, trait aggressiveness, violent media consumption, whether the participant had taken a firearm safety course, whether there were any firearms in the home, attitudes toward guns, and interest in firearms).

For all models, we report incidence rate ratios (IRRs) with SEs, *z* tests, and the corresponding *P* values. *P* values were 2-tailed and considered significant at less than .05. Multivariate models were performed using Stata statistical software version 15.1 (StataCorp). Other analyses were performed using SPSS statistical software version 34 (IBM) and R statistical software version 3.5.1 (R Project for Statistical Computing). Complete details of the analyses are presented in the statistical analysis plan in Supplement 1).

## Results

### Participants

There were 220 participating children (110 pairs) aged 8 to 12 years (mean [SD] age, 9.9 [1.4] years) included in the analysis. There were 163 white participants (74.1%) and 129 boys (58.6%). According to reporting from parents or guardians, 81 participants (36.8%) had at least 1 firearm in the home and 53 participants (24.1%) had taken a firearm safety course. The active players vs passive observers yielded no main or interaction effects in our models.

### Random Assignment Check

Children were not significantly different across conditions on any of the covariates (Table 1). A χ^2^ test found the number of excluded participants did not differ across video game condition (χ^2^_2_ = 1.03; *P* > .59). Thus, random assignment to condition was successful.

**Table 1.** Descriptive Statistics for Participant Covariates Stratified by Video Game Condition

Variable	Condition, No. (%)		
	Nonviolent (n = 70)	Sword Violence (n = 74)	Gun Violence (n = 76)
Sex^a^			
Male	36 (51.4)	48 (64.8)	45 (59.2)
Female^a^	34 (48.6)	26 (35.1)	31 (40.8)
Age, y^a^			
8	14 (20.0)	14 (18.9)	23 (30.3)
9	18 (25.7)	11 (14.9)	10 (13.2)
10	10 (14.3)	24 (32.4)	14 (18.4)
11	16 (22.9)	14 (18.9)	12 (15.8)
12	11 (15.7)	11 (14.9)	17 (22.4)
Mean (SD)	9.9 (1.4)	10.0 (1.3)	9.9 (1.6)
Race/ethnicity^a^			
White	53 (76.8)	54 (73.0)	56 (73.7)
Black	9 (13.0)	8 (10.8)	8 (10.5)
Hispanic	2 (2.9)	3 (4.1)	0
Asian	0	1 (1.4)	2 (2.6)
Other	5 (7.2)	8 (10.8)	10 (13.2)
Trait aggressiveness, mean (SD)^b^	0.70 (0.51)	0.69 (0.43)	0.82 (0.43)
Violent media exposure, mean (SD)^c^	6.44 (1.76)	6.69 (1.97)	6.48 (1.93)
Attitudes toward guns, mean (SD)^d^	3.07 (0.63)	3.02 (0.62)	2.88 (0.59)
Have a firearm in the home^e^			
No	39 (55.7)	49 (67.1)	49 (65.3)
Yes	31 (44.3)	24 (32.9)	26 (34.7)
Predicted interest in firearms^e^^,^^f^			
Not at all interested	19 (27.5)	18 (24.7)	13 (17.1)
Sort of interested	16 (23.2)	12 (16.4)	27 (35.5)
A little interested	16 (23.2)	12 (16.4)	11 (14.5)
Somewhat interested	12 (17.4)	24 (32.9)	18 (23.7)
Very much interested	6 (8.7)	7 (9.6)	7 (9.2)
Mean (SD)	1.6 (1.3)	1.9 (1.4)	1.7 (1.3)
Took firearm safety course^g^	16 (23.2)	17 (24.6)	20 (28.2)

^a^ One parent or guardian did not provide this information on their child. Thus, these numbers are based on 219 rather than 220 participants.

^b^ Range, 0 to 3; higher scores indicate more frequent aggressive behavior.

^c^ Scored by multiplying frequency of engagement with media score (range, 1-3; higher scores indicate greater frequency) and media violence rating score (range, 1-7; higher scores indicating more violent content). Total scores range, 1 to 21.

^d^ Range, 0 to 4; higher scores indicate stricter attitudes toward guns.

^e^ Two parents or guardians did not provide information for their child on predicted interest in firearms. Thus, the numbers are based on 218 rather than 220 participants.

^f^ Range, 0 to 4; higher scores indicate more interest.

^g^ Twenty-nine parents or guardians did not provide information on having taken a firearm safety course. Thus, the numbers are based on 191 rather than 220 participants.

### Game Violence Manipulation Check

As predicted, the participants rated the 2 violent versions (ie, guns and swords) as more violent than the nonviolent version (Table 2). Violence ratings for the gun and sword violence conditions did not differ. The number of monsters killed and the number of chests opened did not differ between the gun and sword violence conditions. The 3 conditions did not differ on the other rating dimensions. Thus, the violence manipulation was successful.

**Table 2.** Comparison of Participants’ Ratings of Video Games in the 3 Video Game Conditions

Assessment	Mean (SE)			*P* Value^a^
	Nonviolent (n = 70)	Sword Violence (n = 74)	Gun Violence (n = 76)	
Played the game before, No. (%)	64 (91.4)	67 (90.5)	69 (90.8)	.98^b^
Seen another play the game before, No. (%)	67 (95.7)	68 (91.9)	70 (92.1)	.60^b^
I am familiar with the game^c^	2.90 (0.17)	2.82 (0.15)	2.89 (0.13)	.92
I liked the game^c^	2.73 (0.16)	2.57 (0.13)	2.46 (0.15)	.43
The game was exciting^c^	2.17 (0.15)	2.18 (0.13)	2.07 (0.15)	.82
The game was boring^c^	2.96 (0.13)	2.99 (0.11)	2.79 (0.15)	.51
The game was fun^c^	2.51 (0.16)	2.42 (0.14)	2.32 (0.15)	.64
I was part of the action^c^	1.94 (0.15)	1.85 (0.15)	1.78 (0.17)	.76
The game was violent^c^	0.47 (0.10)	1.22 (0.13)	1.50 (0.13)	<.001^d^
I want to play more of the game^c^	2.49 (0.18)	2.23 (0.18)	2.17 (0.16)	.39
A friend would want to play more^c^	2.14 (0.16)	2.29 (0.16)	2.45 (0.15)	.39^e^
No. of chests opened^f^	29.71 (2.52)	17.94 (1.81)	15.87 (1.55)	<.001^g^
No. of monsters killed^f^	NA	4.97 (0.78)	4.53 (0.78)	.69^h^

Abbreviation: NA, not applicable.

^a^ *P* values are based on Fisher exact test for count data or 1-way analysis of variance with 2 *df* in the numerator and 217 *df* in the denominator. ^b^*P* value calculated with a χ^2^ test.

^c^ Range, 0 to 4, with 0 indicating not at all and 4, very much.

^d^ Fisher least significant difference test found that the sword and gun violence conditions were significantly more violent than the nonviolent condition but were not significantly different from each other.

^e^ Based on 1-way analysis of variance with 6 *df* in the numerator and 216 *df* in the denominator because of missing values.

^f^ Only half of the participants played the game, so for these values, n = 35 for the nonviolent condition, n = 37 for the sword violence condition, and n = 38 for the gun violence condition.

^g^ Fisher least significant difference test based on 2 *df* in the numerator and 105 *df* in the denominator found that significantly more chests were opened in the nonviolent condition than in the other 2 conditions.

^h^ Based on a 2-tailed *t* test with 1 *df* in the numerator and 72 *df* in the denominator because only two-thirds of the players were able to kill monsters.

### Descriptive Statistics

Of 242 children tested, 220 (90.9%) found the handguns, regardless of condition. The 22 participants (9.1%) who did not find a handgun were excluded from further analyses because they could not handle a handgun or pull the trigger. However, children who found the handgun but did not touch it were included.

Of 220 children who found a handgun, 13 (6.0%) told an adult and did not touch a handgun, 35 (15.9%) told an adult but also touched a handgun, 87 (40.0%) did not touch a handgun but did not tell an adult, and 85 (38.6%) touched a handgun and did not tell an adult. Among the 120 participants who touched a handgun, the mean (SD) time spent holding it was 96.5 (147.7) seconds, and 39 participants (32.5%) pulled the trigger at least once. A total of 966 trigger pulls were recorded, with 346 (35.8%) aimed at the self or the partner. Table 3 presents statistics for the outcomes.

**Table 3.** Statistics for Total Trigger Pulls, Pulling Trigger at Self or Partner, Touching a Handgun, and Seconds Holding a Handgun by Video Game Condition

Outcome	Nonviolent (n = 70)	Sword Violence (n = 74)	Gun Violence (n = 76)	*P* Value^a^
Total trigger pulls				
Mean (SE)	2.14 (0.99)	3.20 (1.13)	7.62 (2.13)	.03^b^
Adjusted median (IQR)	2.99 (1.52)	3.56 (1.81)	10.14 (5.15)	
Trigger pulls at self or partner				
Mean (SE)	0.14 (0.11)	1.41 (0.66)	3.05 (1.04)	.02^c^
Median (IQR)	0.19 (0.06)	1.50 (0.49)	3.44 (1.13)	
Touched handgun, No. (%)	31 (44.3)	42 (56.7)	47 (61.8)	.09^c^
Time handling a handgun, s				
Mean (SE)	25.36 (9.34)	65.74 (17.87)	64.96 (12.43)	.07^d^
Median (IQR)	36.10 (19.44)	71.68 (38.59)	91.49 (49.26)	

Abbreviation: IQR, interquartile range.

^a^ *P* values are based on Fisher exact test for count data or 1-way analysis of variance tests with 2 *df* as the numerator and 217 *df* as the denominator.

^b^ The gun violence condition had significantly more trigger pulls than the other 2 conditions.

^c^ The gun violence condition was significantly different from the nonviolent condition but not the sword violence condition.

^d^ The nonviolent condition was significantly lower than the other 2 conditions.

### Outliers

Because outliers can have an undue influence on statistical results, we examined the data for extreme outliers. We eliminated 1 pair who was more than 5 SDs from the mean for both time spent holding a handgun and trigger pulls. The coders also recommended eliminating another pair because of unusual and extremely aggressive behavior.

### Touched a Handgun

Of 220 children who found a handgun, 61.8% (47 of 76) in the gun violence condition, 56.8% (42 of 74) in the sword condition, and 44.3% (31 of 70) in the control condition touched a handgun. The difference across conditions was nonsignificant (χ^2^_2_ = 4.75; *P* = .09).

### Time Spent Holding a Handgun

In the reduced model, the gun violence condition increased time spent holding a handgun, although the effect was nonsignificant (*z* = 1.75; *P* = .08). The ordering of medians across conditions was in the predicted direction (ie, 91.49 seconds in the gun violence condition, 71.68 seconds in the sword violence condition, and 36.10 seconds in the nonviolent control condition). In the full model, differences in time spend holding a handgun were nonsignificant for the gun (*z* = 1.55; *P* = 12) and sword (*z* = 1.64; *P* = .10) violence conditions.

### Trigger Pulls

In the reduced model, participants in the gun violence condition pulled the trigger more times than participants in other conditions, although the effect was nonsignificant (*z* = 1.66; *P* = .10). The ordering of medians across conditions was in the predicted direction (ie, 10.14 pulls in the gun violence condition, 3.56 pulls in the sword violence condition, and 2.99 pulls in the nonviolent control condition). In the full model, the difference between the gun and sword violence conditions was nonsignificant.

In the reduced model, participants in the violent game conditions pulled the trigger at themselves or their partner more than participants in the nonviolent condition. The effect was significant for the gun violence condition (IRR, 18.36; 95% CI, 2.21-152.51) but nonsignificant for the sword condition. The ordering of medians across conditions was in the predicted direction (ie, 3.44 pulls in the gun violence condition, 1.50 pulls in the sword violence condition, and 0.19 pulls in the nonviolent control condition). In the full model, the gun (IRR, 6.89; 95% CI, 1.15-41.23) and the sword (IRR, 12.23; 95% CI, 1.90-78.70) violence conditions were statistically significant.

### Covariates

Table 4 presents the results of negative binomial regression models for the covariates. Self-reported exposure to violent media was positively associated with total trigger pulls (IRR, 1.40; 95% CI, 1.00-1.98) and trigger pulls at oneself or one’s partner (IRR, 1.88; 95% CI, 1.29-2.72). Trait aggression was positively associated with total trigger pulls (IRR, 13.52; 95% CI, 3.14-58.29), trigger pulls at oneself or one’s partner (IRR, 25.69; 95% CI, 5.92-111.39), and time spent holding a handgun (IRR, 4.22; 95% CI, 1.62-11.02). Age was negatively associated with trigger pulls at oneself or one’s partner (IRR, 0.68; 95% CI, 0.52-0.88) and time spent holding a handgun (IRR, 0.74; 95% CI, 0.58-0.95). Predicted interest in guns was positively associated with total trigger pulls (IRR, 2.83; 95% CI, 1.79-4.47), trigger pulls at oneself or one’s partner (IRR, 2.75; 95% CI, 1.87-4.03), and time spent holding a handgun (IRR, 1.65; 95% CI, 1.22-2.23). Having a stricter attitude toward guns was negatively associated with total trigger pulls (IRR, 0.27; 95% CI, 0.10-0.74) and time spent holding a handgun (IRR, 0.40; 95% CI, 0.20-0.82). Children with at least 1 gun in the home were less likely to pull the trigger at themselves or their partners (IRR, 0.05; 95% CI, 0.01-0.38). Children who had taken a firearm safety course pulled the trigger fewer times (IRR, 0.15; 95% CI, 0.03-0.80) and spent less time holding a handgun (IRR, 0.10; 95% CI, 0.03-0.35).

**Table 4.** Negative Binomial Regression Models^a^

Parameter	Incidence Rate Ratio (95% CI)					
	Trigger Pulls		Trigger Pulls at Self or Other		Time With Handgun	
	Model 1	Model 2	Model 3	Model 4	Model 5	Model 6
Nonviolent condition	1.47 (0.37-5.84)	1.66 (0.03-94.62)	0.13 (0.02-0.96)	0 (0-0.17)	16.66 (6.88-40.34)	581.38 (17.82-18 971.73)
Gun violence condition	3.39 (0.80-14.29)	2.36 (0.40-13.77)	18.36 (2.21-152.51)	6.89 (1.15-41.23)	2.53 (0.90-7.17)	2.56 (0.78-8.37)
Sword violence condition	1.19 (0.26-5.39)	2.45 (0.36-16.78)	8.01 (0.89-71.97)	12.23 (1.90-78.70)	1.99 (0.64-6.14)	2.99 (0.81-11.10)
Sex	2.03 (0.83-4.99)	0.65 (0.19-2.16)	1.49 (0.69-3.24)	0.75 (0.25-2.26)	2.17 (1.07-4.38)	1.12 (0.57-2.22)
Attitude toward guns	NA	0.27 (0.10-0.74)	NA	0.81 (0.29-2.26)	NA	0.40 (0.20-0.82)
Trait aggression	NA	13.52 (3.14-58.29)	NA	25.69 (5.92-111.39)	NA	4.22 (1.62-11.02)
Violent media exposure	NA	1.40 (1.00-1.98)	NA	1.88 (1.29-2.72)	NA	1.04 (0.84-1.28)
Predicted interest in guns	NA	2.83 (1.79-4.47)	NA	2.75 (1.87-4.03)	NA	1.65 (1.22-2.23)
Age	NA	0.80 (0.59-1.09)	NA	0.68 (0.52-0.88)	NA	0.74 (0.58-0.95)
Took a firearm safety course	NA	0.15 (0.03-0.80)	NA	3.27 (0.38-22.33)	NA	0.10 (0.03-0.35)
Any guns in the home	NA	0.84 (0.14-5.06)	NA	0.05 (0.01-0.38)	NA	1.76 (0.63-4.93)

Abbreviation: NA, not applicable.

^a^ Models 1, 3, and 5 are reduced models, and models 2, 4, and 6 are full models.

## Discussion

In this study, playing a violent video game increased the likelihood that children would touch a real handgun, increased time spent holding a handgun, and increased pulling the trigger at oneself and others. It did not influence total trigger pulls. While each of the outcomes point in the predicted direction, some were nonsignificant, perhaps because of insufficient statistical power. Compared with exposure to sword violence, exposure to gun violence produced larger effects in time spent holding a handgun and possibly pulling the trigger at oneself or others, suggesting that exposure to gun violence is different than exposure to other types of violence. This may be because media violence with a gun more easily translates onto handling and shooting a real gun compared with other types of violence. These results are consistent with social learning theory^5^ in that using a gun in the video game yielded positive outcomes (ie, killing hostile monsters and opening more chests). Effects were not larger for video game players than for watchers, suggesting that modeling may be the underlying mechanism for the observed effects.

This study partially replicates the findings of the 2017 movie study by Dillon and Bushman.^7^ In addition, it shows that these effects are not bound by a single medium and that they can occur with unrealistic depictions of firearms and with nonhuman targets (ie, monsters). This study also found long-term effects of exposure to media violence—children who habitually consumed violent media were more likely to shoot at themselves or their partners independent of condition. Thus, although a single exposure to video game violence may only have short-term effects on dangerous behavior around guns, habitual exposure to violent media could also be a risk factor. The findings were robust to demographic characteristics. Short-term and long-term effects were found for boys and girls, regardless of whether they played or watched the game.

Other risk factors can also influence whether children engage in dangerous behavior around real guns. We controlled for some likely risk factors (ie, sex, age, trait aggressiveness, violent media consumption, whether the participant had taken a firearm safety course, firearms in the home, attitudes toward guns, and interest in firearms), but there are many others we did not examine (eg, developmental risk factors, low IQ score, living in a high-crime neighborhood, emotion regulation difficulties, impulsiveness, risk-taking tendencies).

We found that having taken firearm safety courses reduced some risky behavior around handguns, although a 2002 study^16^ on the efficacy of these courses had mixed results. The results of this study were also mixed because children who had taken a firearm safety course pulled the trigger more times while pointing a handgun at themselves or their partner, although the effect was nonsignificant. Additionally, children with guns in the home were less likely to engage in dangerous behaviors, which may reflect gun-owning parents or guardians teaching their children principles of firearm safety.

### Limitations

This study had limitations. Although we tried to mimic a real-world setting (ie, finding a gun hidden in a drawer), this study was conducted in an artificial setting—a university laboratory. We predict even stronger effects might have been observed in more realistic settings for a variety of reasons. For example, participants might feel less inhibited in a home setting than in a laboratory setting, owing to both familiarity and to not being monitored. In addition, some participants thought the handguns were fake, stating that a researcher would not leave a real gun in a university laboratory. Future research should replicate these findings in a more natural setting.

Another limitation is that participants spanned a large and developmentally important age range. Age was significantly related to 2 outcome measures. However, the ages did not differ across conditions.

Another limitation is that the *Minecraft* video game we used was not very violent (eg, crude graphics, no blood and gore). Violence ratings for the violent conditions were lower than 2 on a scale of 0 to 4 (Table 2). For ethical reasons, we could not use a more graphically violent video game, although 31% of children’s favorite games listed were rated T for teenagers 13 years and older and 30% were rated M for mature players 17 years and older. In addition, the player’s avatar was neither clearly visible nor customizable, which may have diminished identification with the avatar. Also, the relatively short duration of gameplay may not have given time to reinforce learning via direct experience, potentially explaining the lack of differences between players and watchers. A more violent game might yield stronger results.

An additional limitation was using self-report data for several variables. It would be useful for future research to obtain reports from others (eg, parents, teachers, peers).

## Conclusions

It is well established that consumption of violent media increases aggression in children in both the short and long term.^17^ The danger of violent media exposure has been compared with the danger of exposure to real-world violence,^18^ and some studies^19^ found effect sizes large enough to consider media violence a public health threat. Our study highlights another danger of violent media exposure: it increases dangerous behavior around firearms. Specifically, exposure to violent video games can increase a child’s interest in firearms, including shooting a handgun at themselves or others. In addition, habitual exposure to violent media was a risk factor for dangerous behavior around real guns. As such, parents and guardians should be cognizant of the risk associated with exposure to violent media. Most importantly, gun owners should secure their firearms.

## Notes

Supplement 1. **Trial Protocol and Statistical Analysis Plan**

Supplement 2. **eAppendix.** Games and Guns Code and Output

Supplement 3. **Data Sharing Statement**
