# Error in Results and in Table 3

**DOI:** 10.1001/jamanetworkopen.2019.7377

**Published:** 2019-06-28

In the Original Investigation titled “Effect of Exposure to Gun Violence in Video Games on Children’s Dangerous Behavior With Real Guns: A Randomized Clinical Trial,”^1^ published May 31, 2019, the medians provided in the Time Spent Holding a Handgun and Trigger Pulls subsections of the Results section should have been described as adjusted medians. In Table 3, all the means (SEs) and medians (interquartile ranges) should have been labeled as adjusted. This article has been corrected.^1^
