# Supplementary material for: Effect of Exposure to Gun Violence in Video Games on Children’s Dangerous Behavior With Real Guns: A Randomized Clinical Trial
Source: JAMA Netw Open. 2019 May 31;2(5):e194319. doi: 10.1001/jamanetworkopen.2019.4319 (PMC6547242; doi:10.1001/jamanetworkopen.2019.4319)
Supplement: Supplement 1. — Trial Protocol and Statistical Analysis Plan [file jamanetwopen-2-e194319-s001.pdf]

Study Protocol

Objectives

This research tests the effects of the mere presence of guns in the mass media (e.g. movies, video games, TV programs) on children. We predict that seeing guns in movies will increase subsequent aggression, and will lead to more positive attitudes about guns.

Background and Rationale

On 14 December 2012, after shooting his mother, 20-year-old Adam Lanza shot and killed 20 children and 6 employees in Sandy Hook elementary school, in Newtown, Connecticut. In the wake of the Newton shootings, there has been considerable discussion about gun violence. What is conspicuously absent from these discussions, however, is the fact that just *SEEING* guns can increase aggression.[i] This effect, called the "weapons effect," has been replicated in over 50 other studies.[ii] The effect occurs for angry and non-angry individuals, both inside and outside the lab.

A recent analysis of top selling films found that the depiction of guns in violent scenes in PG-13 films that target youth has increased from the level of G and PG files in 1985 when the rating was introduced, to the level of R films by 2005, to exceed the level of R films since 2012.[iii] By definition, a PG-13 movie is supposed to have less violence than an R-rated movie. The Motion Picture Association of America says on its website that the violence in a PG-13 movie "does not reach the restricted R category." Our study shows that it does. By including guns in violent scenes, film producers may be inadvertently increasing aggression in youth via a weapons effect. Experiment 1 directly tests this hypothesis. Previous research has shown that when exposed to movie characters that smoke, many youth are more likely to start smoking themselves[iv]; the same is true for characters that drink[v]. Similarly, we predict that youth will be more interested in acquiring and using guns after exposure to gun violence in films. We also predict they will behave more aggressively following exposure to guns in movies.

Experiment

Participants will be children 8-12 years old. Children will be tested in pairs. Each child will bring a sibling, relative, or friend to the study. Participants and partners will each receive a $25 gift card. The design is a 2 (watch vs. play) by 3 (non-violent, violent with swords, violent with guns) between-subjects design. The outcome variables are the time spent handing a gun (in seconds), the number of times the trigger is pulled, and the number of times the trigger is pulled while pointing the handgun at oneself or another. After filling out a pre-test survey measuring trait aggression and media diet, one participant will be randomly assigned to play a video game while the other watches. The video game participants will play is a modded version of *Minecraft*. The participant pairs will be randomly assigned to one of three conditions: a non-violent condition with no weapons and no monsters, a violent condition with swords and monsters, and a violent condition with guns and monsters. Participants will play for 20 minutes, after which they will fill out a survey measuring their familiarity with *Minecraft* and their opinion toward the game.

After completing the survey, participants will be placed in a room containing games and toys, with hidden cameras. Participants will be told that they can play with any of the games and toys in the room for the next 20 minutes. The toys and games will be in two drawers of a cabinet, while the bottommost drawer will contain two Sig Sauer P250 handguns. The handguns have been disabled and are unable to fire. In addition, a counter is placed inside the magazine well to count the number of times the trigger is depressed with enough force to discharge the handgun. This allows us to distinguish reliably the children who pull the trigger from those who handle the gun but do not pull the trigger. Parents will be asked to

predict whether their child will handle the real gun and pull the trigger. The researcher and the parents will be able to watch the session via a monitor in a control room. After 20 minutes, the participants will fill out a survey on their attitudes toward guns. A thorough debriefing will follow.

We predict that participants who play the game with guns will handle the gun longer, pull the trigger more times, and point the gun at oneself or another and pull the trigger more times than participants in the other conditions. One week after a child participates in Experiment 3, the researcher will call or email the parent to follow up.

Attitudes Toward Guns
To measure participants' attitudes toward guns, we will use the 15-item Attitudes Towards Guns and Violence Questionnaire (see Appendix C)[vi], which has been validated on children 6-17 years old and young adults.[vii] It contains three subscales: *Comfort With Aggression* (e.g., "I don't like being around people with guns because someone could end up getting hurt" and "I wish everyone would get rid of all their guns"), *Excitement* (e.g., "I bet it would feel really cool to walk down the street with a gun in my pocket" and "I think it would be fun to play around with a real gun"), and *Power/Safety* (e.g., "Carrying a gun makes people feel powerful and strong" and "Carrying a gun makes people feel safe"). Items are scored using a 5-point scale (1=*Strongly disagree* to 5=*Strongly agree*). We will also measure whether the child has ever fired a gun, and whether the child has taken a gun safety class.

Prior to the experiment, parents will rate their child's level of interest in real guns using a 5-point scale (1=*no interest* to 5=*very interested*), as in previous research.[viii]

Guns in the Home
We will record the number of guns in the home. If there are guns in the home, we will record what types of guns they are, whether any guns are loaded, and whether any guns are locked up.

Behavioral Measure of Aggression
Experiments 1 and 2 will use a measure of aggression that has been successfully used for children about this age in our previous research.[ix, x] Participants will complete a 25-trial competitive reaction time task with an ostensible partner of the same sex in which the winner can blast the loser with loud noise through headphones. The noise is a mixture of noises that most people hate (e.g., fingernails scratching a chalkboard, dentist drills, sirens). The noise levels range from *Level 1*=60 decibels to *Level 10*=105 decibels (about the same level as a fire alarm). Participants will be told that lose levels 8, 9, 10 can cause "permanent hearing damage to their partner." [xi] The noise levels are well within Occupational Safety and Health Administration (OSHA) safety levels (i.e.,120 decibels). The threshold of pain for the human ear is 140 decibels. Because decibels are measured on a logarithm scale, level 105 decibels is unpleasant but not painful or harmful. A non-aggressive no-noise option (*Level 0*) is also provided. The winner can also determine the duration of the loser's suffering by controlling the noise duration (*Level 1*=0.5 sec to *Level 10*=5 sec). The participant wins half of the trials (randomly determined). The ostensible partner set random noise intensities and durations across the 25 trials. Basically, within the ethical limits of the laboratory, participants control a weapon that can be used to blast their partner with unpleasant noise. A through debriefing will follow that we have successfully used in previous studies in which children are exposed to violent media. The parent will also be present during the debriefing. The researcher will read the debriefing to the child and answer any questions he or she has.

For each of the dependent variables, we predict a main effect for violent content, a main effect for the presence of guns, and an interaction between violent content and the presence of guns. Specifically, we

predict the highest levels of pro-gun attitudes and aggression among participants who see a violent clip containing guns.

Power Analysis

A power analyses revealed that 64 participants per group are needed to detect a medium sized effect at the .05 significance level with power=.80. Thus, the experiment requires 192 participants

We aim to use ResearchMatch.org, a national electronic, web-based recruitment tool created through the Clinical & Translational Science Awards Consortium in 2009 and is maintained at Vanderbilt University (see ResearchMatch General Description.pdf). The Vanderbilt IRB provides oversight for ResearchMatch as a recruitment tool, and locally is managed by Rose Hallarn and the Center for Clinical & Translational Research at OSUMC. Individuals in the Columbus area who have self-identified or have identified their children as interested in participating in research studies will be identified through a targeted search using approved inclusion criteria (ages 8-12yo, willing to travel to the Ohio State University campus). A feasibility search using ResearchMatch with these criteria yielded 90-100 adolescents available for recruitment. Once approved, investigators will send a message to identified individuals about the available study (see ResearchMatch Recruitment Message.docx). We will also recruit participants through other means (e.g., ads placed on craigslist, Facebook, newspapers)

Internal Validity

Because all studies are laboratory experiments, threats to internal validity are low.

Statistical Analysis Plan

Data will be collected in three ways: parental consent is collected directly by Qualtrics; the participants' survey responses are done by hand on paper, and the participants' handling of the handguns is recorded as video. After the conclusion of the study, the paper responses will be uploaded by the research staff into a Qualtrics survey. The videos will be content analyzed by trained research assistants who observe the participants' behavior, verify the trigger pull count, and note if the trigger is depressed while the handguns are pointed at oneself or the other participant. At this point, the data can be merged into a single dataframe for validation and cleaning. Cleaning will be done in Excel and R.

Once the data has been cleaned, the variables will each be examined via summary statistics and visually. If necessary, variables will be transformed to conform to the assumptions of the statistical tests, the Cronbach's alpha of the survey batteries will be calculated, potential outliers will be identified, and preliminary bivariate and chi-squared tests will be run on the outcome variables. In addition, the three conditions will be tested for differences in the subjects along the measured variables.

After this, the dataframe will be imported into STATA to perform a generalized estimated equation (GEE) with an exchangeable correlation structure was used to analyze data, with pairs as unit of analysis. All outcome variables will be measured with a negative binomial log-link model. First, each outcome variable will be tested with the condition as the sole predictor variable. Next, each control variable (trait aggression, attitude toward guns, etc.) will be tested along with the condition. Finally, the full model will include condition and all of the control variables. The research team will find the model of best fit, though only the reduced (condition only) and full models (condition, gender, age, whether the participant had taken a firearm safety course, if there is a firearm in the home, attitudes toward guns, trait aggression, consumption of violent media, parental estimation of child's interest in firearms) will be reported.

In addition, data on the participants' familiarity with, opinion of, and performance in *Minecraft* will be recorded, primarily to verify that the participants don't significantly differ between conditions. However, exploratory testing will be done to determine if these variables also have a significant impact on the outcome variables. Whether the participant played or watched the game will also be tested.

---

[i] Bushman, B. J. (in press). The weapons effect. *JAMA Pediatrics*.

[ii] Carlson, M., Marcus-Newhall, A., & Miller, N. (1990). Effects of situational aggression cues: A quantitative review. *Journal of Personality and Social Psychology, 58*, 622–633.

[iii] Bushman, B. J., Jamieson, P. E., Weitz, I., & Romer, D. (in press). Gun violence trends in movies. *Pediatrics*

[iv] Dal Cin, S., Stoolmiller, M., & Sargent, J. D. (2012). When movies matter: Exposure to smoking in movies and changes in smoking behavior. *Journal of Health Communication, 17*(1), 76-89.

[v] Wills, T. A., Sargent, J. D., Gibbons, F. X., Gerrard, M., & Stoolmiller, M. (2009). Movie exposure to alcohol cues and adolescent alcohol problems: a longitudinal analysis in a national sample. *Psychology of Addictive Behaviors*, 23(1). 23-25.

[vi] Shapiro, J. P., Dorman, R. L., Burkey, W. M., Welker, C. J., & Clough, J. B. (1997). Development and factor analysis of a measure of youth attitudes towards guns and violence. *Journal of Clinical Child Psychology, 26* (93), 311-320.

[vii] Shapiro, J. P. (2000). Attitudes Toward Guns and Violence Questionnaire: Manual. Los Angeles, CA: Western Psychological Services.

[viii] Jackman, G. A., Farah, M. M., Kellerman, A. L., & Simon, H. K. (2001). Seeing is believing: What do boys do when they find a real gun? *Pediatrics, 107*(6), 1247-1250. DOI: 10.1542/peds.107.6.1247

[ix] Konijn, E. A., Nije Bijvank, M., & Bushman, B. J. (2007). I wish I were a warrior: The role of wishful identification in effects of violent video games on aggression in adolescent boys. *Developmental Psychology, 43*(4), 1038-1044. doi: 10.1037/0012-1649.43.4.1038

[x] Gabbiadini, A., Riva, P., Andrighetto, L., Volpato, C., & Bushman, B. J. (in press). Moral disengagement moderates the effect of violent video games on self-control, cheating and aggression. *Social Psychological and Personality Science*.

[xi] Konijn, E. A., Nije Bijvank, M., & Bushman, B. J. (2007). I wish I were a warrior: The role of wishful identification in effects of violent video games on aggression in adolescent boys. *Developmental Psychology, 43*(4), 1038-1044. doi: 10.1037/0012-1649.43.4.1038
