# Supplementary material for: Effect of Exposure to Gun Violence in Video Games on Children’s Dangerous Behavior With Real Guns: A Randomized Clinical Trial
Source: JAMA Netw Open. 2019 May 31;2(5):e194319. doi: 10.1001/jamanetworkopen.2019.4319 (PMC6547242; doi:10.1001/jamanetworkopen.2019.4319)
Supplement: Supplement 2. — eAppendix. Games and Guns Code and Output [file jamanetwopen-2-e194319-s002.pdf]

Supplementary Online Content

Chang JH, Bushman BJ. Effect of exposure to gun violence in video games on children's dangerous behavior with real guns: a randomized clinical trial. *JAMA Netw Open*. 2019;2(5):e194319. doi:10.1001/jamanetworkopen.2019.4319

eAppendix. Games and Guns code and output

This supplementary material has been provided by the authors to give readers additional information about their work.

© 2019 Chang JH et al. *JAMA Network Open.*

eAppendix. Games and Guns code and output

Notes:

- The CSV provided, "Games and Guns trim.csv", is the dataset including all participants who were not excluded due to ineligibility or outliers. This includes participants who did not find the handguns. The majority of the analyses here trims off the participants who did not find the handguns, done as follows:
    - ggr <- as.data.frame(gg.trim[gg.trim$FoundGun ==1,])
- Note that the condition variable ($Cond) should be specified as a factor
- Libraries used are:
    - library(fifer)
    - library(agricolae)
    - library(psych)
    - library(plotrix)
- Some trivial calculations, like percentages, aren't included in the code here
- The models run in STATA were done using the dataframe excluding those who did not find the gun (ggr)

Table 1

Sex by condition

```
> table(ggr$Sex, ggr$Cond)

     1  2  3
  0 34 26 31
  1 36 48 45

> table1 <- table(ggr$Cond, ggr$Sex)
> chisq.post.hoc(table1, test='chisq.test')
Adjusted p-values used the fdr method.

  comparison  raw.p  adj.p
1    1 vs. 2 0.1428 0.4284
2    1 vs. 3 0.4363 0.5857
3    2 vs. 3 0.5857 0.5857

> reg1 <- aov(Sex ~ Cond, data = ggr)
> summary(reg1)
             Df Sum Sq Mean Sq F value Pr(>F)
```

© 2019 Chang JH et al. *JAMA Network Open.*

```
Cond          2   0.65  0.3266   1.345  0.263
Residuals   217  52.71  0.2429
```

Age by condition, mean, and SD

```
> table(ggr$Age, ggr$Cond)

      1  2  3
  8  14 14 23
  9  18 11 10
  10 10 24 14
  11 16 14 12
  12 11 11 17

> table1 <- table(ggr$Cond, ggr$Age)
> chisq.post.hoc(table1, test='chisq.test')

Adjusted p-values used the fdr method.

  comparison  raw.p adj.p
1    1 vs. 2 0.1152 0.179
2    1 vs. 3 0.1540 0.179
3    2 vs. 3 0.1790 0.179

> mean(na.omit(ggr$Age[ggr$Cond == 1]))
[1] 9.884058
> mean(na.omit(ggr$Age[ggr$Cond == 2]))
[1] 9.959459
> mean(na.omit(ggr$Age[ggr$Cond == 3]))
[1] 9.868421

> sd(na.omit(ggr$Age[ggr$Cond == 1]))
[1] 1.39891
> sd(na.omit(ggr$Age[ggr$Cond == 2]))
[1] 1.307923
> sd(na.omit(ggr$Age[ggr$Cond == 3]))
[1] 1.552135

> reg1 <- aov(Age ~ Cond, data = ggr)
> summary(reg1)
             Df Sum Sq Mean Sq F value Pr(>F)
```

© 2019 Chang JH et al. *JAMA Network Open.*

```
Cond           2    0.4  0.1756   0.086  0.917
Residuals   216  438.6  2.0307
1 observation deleted due to missingness

> std.error(na.omit(ggr$Age[ggr$Cond == 1]))
[1] 0.1684089
> std.error(na.omit(ggr$Age[ggr$Cond == 2]))
[1] 0.152043
> std.error(na.omit(ggr$Age[ggr$Cond == 3]))
[1] 0.1780421
```

Race by condition

```
table(ggr$Race, ggr$Cond)

     1  2  3
  1  0  1  2
  2  9  8  8
  3  2  3  0
  4 53 54 56
  5  5  8 10

> table1 <- table(ggr$Cond, ggr$Race)
> chisq.post.hoc(table1, test='chisq.test')

Adjusted p-values used the fdr method.

  comparison  raw.p  adj.p
1    1 vs. 2 0.7747 0.7747
2    1 vs. 3 0.2412 0.7019
3    2 vs. 3 0.4679 0.7019

> reg1 <- aov(Race ~ Cond, data = ggr)
> summary(reg1)
             Df Sum Sq Mean Sq F value Pr(>F)
Cond          2   0.13  0.0642   0.094   0.91
Residuals   216 147.20  0.6815
1 observation deleted due to missingness
```

Trait aggression by condition, mean, and SD

```
> reg1 <- aov(BehMean ~ Cond, data = ggr)
```

© 2019 Chang JH et al. *JAMA Network Open.*

```
> summary(reg1)
             Df Sum Sq Mean Sq F value Pr(>F)
Cond          2   0.79  0.3958   1.895  0.153
Residuals   217  45.31  0.2088

> out1 <- LSD.test(reg1, "Cond", p.adj = "none")
> out1

$`statistics`
    MSerror  Df      Mean       CV
  0.2087967 217 0.7384921 61.87511

$parameters
        test p.ajusted name.t ntr alpha
  Fisher-LSD      none   Cond   3  0.05

$means
    BehMean       std  r       LCL       UCL   Min      Max       Q25       Q50       Q75
1 0.7019274 0.5072908 70 0.5942834 0.8095714 0.000 2.111111 0.2708333 0.6250000 0.8888889
2 0.6886261 0.4328174 74 0.5839318 0.7933204 0.000 2.125000 0.3750000 0.5902778 0.8888889
3 0.8207237 0.4301472 76 0.7174161 0.9240312 0.125 2.500000 0.5000000 0.7638889 1.0000000

$comparison
NULL

$groups
    BehMean groups
3 0.8207237      a
1 0.7019274      a
2 0.6886261      a

attr(,"class")
[1] "group"

> std.error(na.omit(ggr$BehMean[ggr$Cond == 1]))
[1] 0.06063285
> std.error(na.omit(ggr$BehMean[ggr$Cond == 2]))
[1] 0.050314
> std.error(na.omit(ggr$BehMean[ggr$Cond == 3]))
[1] 0.04934127
```

© 2019 Chang JH et al. *JAMA Network Open.*

Violent media exposure by condition, mean, and SD

```
> reg1 <- aov(MediaExpS ~ Cond, data = ggr)
> summary(reg1)
             Df Sum Sq Mean Sq F value Pr(>F)
Cond          2    2.7   1.328   0.371  0.691
Residuals   217  777.0   3.580

> out1 <- LSD.test(reg1, "Cond", p.adj = "none")
> out1

$`statistics`
   MSerror  Df     Mean       CV
  3.580468 217 6.537626 28.94342

$parameters
        test p.ajusted name.t ntr alpha
  Fisher-LSD      none   Cond   3  0.05

$means
  MediaExpS      std  r      LCL      UCL      Min      Max      Q25      Q50      Q75
1  6.442063 1.759503 70 5.996307 6.887820 2.777778 10.44444 5.027778 6.222222 7.861111
2  6.690691 1.967564 74 6.257149 7.124233 3.333333 11.88889 5.250000 6.500000 7.750000
3  6.476608 1.934743 76 6.048809 6.904408 2.555556 12.22222 5.194444 6.472222 7.472222

$comparison
NULL

$groups
  MediaExpS groups
2  6.690691      a
3  6.476608      a
1  6.442063      a

attr(,"class")
[1] "group"

> std.error(na.omit(ggr$MediaExpS[ggr$Cond == 1]))
[1] 0.2103009
> std.error(na.omit(ggr$MediaExpS[ggr$Cond == 2]))
[1] 0.2287246
```

© 2019 Chang JH et al. *JAMA Network Open.*

```
> std.error(na.omit(ggr$MediaExpS[ggr$Cond == 3]))
[1] 0.2219303
```

Attitude toward guns by condition, mean, and SD

```
> reg1 <- aov(GunQMn ~ Cond, data = ggr)
> summary(reg1)
             Df Sum Sq Mean Sq F value Pr(>F)  
Cond          1   1.38   1.382   3.705 0.0556 .
Residuals   215  80.19   0.373                 
---
Signif. codes:  0 ‘***’ 0.001 ‘**’ 0.01 ‘*’ 0.05 ‘.’ 0.1 ‘ ’ 1
3 observations deleted due to missingness

> out1 <- LSD.test(reg1, "Cond", p.adj = "none")
> out1

$`statistics`
    MSerror  Df     Mean       CV
  0.3729884 215 2.986175 20.45184

$parameters
        test p.ajusted name.t ntr alpha
  Fisher-LSD      none   Cond   3  0.05

$means
    GunQMn       std  r      LCL      UCL      Min      Max      Q25
1 3.070340 0.6306536 70 2.926461 3.214219 1.733333 4.000000 2.683333
2 3.019048 0.6168185 72 2.877181 3.160915 1.600000 4.000000 2.666667
3 2.876063 0.5885790 75 2.737063 3.015064 1.266667 3.933333 2.433333
       Q50      Q75
1 3.200000 3.600000
2 3.000000 3.550000
3 2.933333 3.333333

$comparison
NULL

$groups
    GunQMn groups
1 3.070340      a
2 3.019048      a
```

© 2019 Chang JH et al. *JAMA Network Open.*

```
3 2.876063      a

attr(,"class")
[1] "group"

> std.error(na.omit(ggr$GunQMn[ggr$Cond == 1]))
[1] 0.07537753
> std.error(na.omit(ggr$GunQMn[ggr$Cond == 2]))
[1] 0.07269275
> std.error(na.omit(ggr$GunQMn[ggr$Cond == 3]))
[1] 0.06796324
```

Guns in household by condition

```
> ggr$HaveGun <- ifelse(ggr$NumGuns > 0, 1, 0)
> table(ggr$HaveGun, ggr$Cond)

     1  2  3
  0 39 49 49
  1 31 24 26

> table1 <- table(ggr$Cond, ggr$HaveGun)
> chisq.post.hoc(table1, test='chisq.test')
Adjusted p-values used the fdr method.

  comparison  raw.p  adj.p
1    1 vs. 2 0.2187 0.4652
2    1 vs. 3 0.3102 0.4652
3    2 vs. 3 0.9550 0.9550
```

Predicted interest in firearms by condition

```
> table(ggr$Cond, ggr$GunInt)

     0  1  2  3  4
  1 19 16 16 12  6
  2 18 12 12 24  7
  3 13 27 11 18  7

> table1 <- table(ggr$Cond, ggr$GunInt)
> chisq.post.hoc(table1, simulate.p.value = TRUE, test='chisq.test')
```

© 2019 Chang JH et al. *JAMA Network Open.*

```
Adjusted p-values used the fdr method.

  comparison  raw.p  adj.p
1    1 vs. 2 0.4178 0.4178
2    1 vs. 3 0.2064 0.3096
3    2 vs. 3 0.1124 0.3096

> reg1 <- aov(as.numeric(GunInt) ~ Cond, data = ggr)
> summary(reg1)

             Df Sum Sq Mean Sq F value Pr(>F)
Cond          2    3.1   1.574   0.917  0.401
Residuals   215  368.8   1.715
2 observations deleted due to missingness

> mean(na.omit(ggr$GunInt[ggr$Cond == 1]))
[1] 1.565217
> mean(na.omit(ggr$GunInt[ggr$Cond == 2]))
[1] 1.863014
> mean(na.omit(ggr$GunInt[ggr$Cond == 3]))
[1] 1.723684
> sd(na.omit(ggr$GunInt[ggr$Cond == 1]))
[1] 1.300207
> sd(na.omit(ggr$GunInt[ggr$Cond == 2]))
[1] 1.367429
> sd(na.omit(ggr$GunInt[ggr$Cond == 3]))
[1] 1.260674

> std.error(na.omit(ggr$GunInt[ggr$Cond == 1]))
[1] 0.1565265
> std.error(na.omit(ggr$GunInt[ggr$Cond == 2]))
[1] 0.1600455
> std.error(na.omit(ggr$GunInt[ggr$Cond == 3]))
[1] 0.1446092
```

Taken gun safety course by condition

```
> table(ggr$GunSafety, ggr$Cond)
```

© 2019 Chang JH et al. *JAMA Network Open.*

```
    1  2  3
  0 53 52 51
  1 16 17 20
>
> table1 <- table(ggr$Cond, ggr$GunSafety)
> chisq.post.hoc(table1, simulate.p.value = TRUE, test='chisq.test')
Adjusted p-values used the fdr method.

  comparison  raw.p  adj.p
1    1 vs. 2 0.2999 0.4498
2    1 vs. 3 0.2734 0.4498
3    2 vs. 3 0.9190 0.9190

> reg1 <- aov(GunSafety ~ Cond, data = ggr)
> summary(reg1)

             Df Sum Sq Mean Sq F value Pr(>F)
Cond          2   0.09 0.04608   0.241  0.786
Residuals   206  39.47 0.19159
11 observations deleted due to missingness
```

© 2019 Chang JH et al. *JAMA Network Open.*

Table 2

Played game before

```
> table(ggr$SeenMov, ggr$Cond)

     1  2  3
  0  6  7  7
  1 64 67 69

> reg1 <- aov(as.integer(SeenMov) ~ Cond, data = ggr)
> summary(reg1)
             Df Sum Sq Mean Sq F value Pr(>F)
Cond          2  0.003 0.00150   0.018  0.982
Residuals   217 18.179 0.08377

> out1 <- LSD.test(reg1, "Cond", p.adj = "none")
> out1

$`statistics`
     MSerror  Df      Mean       CV
  0.08377334 217 0.9090909 31.83799

$parameters
        test p.ajusted name.t ntr alpha
  Fisher-LSD      none   Cond   3  0.05

$means
  as.integer(SeenMov)       std  r       LCL       UCL Min Max Q25 Q50 Q75
1           0.9142857 0.2819630 70 0.8461020 0.9824695   0   1   1   1   1
2           0.9054054 0.2946518 74 0.8390901 0.9717208   0   1   1   1   1
3           0.9078947 0.2910959 76 0.8424578 0.9733317   0   1   1   1   1

$comparison
NULL

$groups
  as.integer(SeenMov) groups
1           0.9142857      a
3           0.9078947      a
2           0.9054054      a
```

© 2019 Chang JH et al. *JAMA Network Open.*

```
attr(,"class")
[1] "group"
```

Seen other play game before

```
> table(ggr$SeenOther, ggr$Cond)

     1  2  3
  0  3  6  6
  1 67 68 70
>
> reg1 <- aov(as.integer(SeenOther) ~ Cond, data = ggr)
> summary(reg1)

             Df Sum Sq Mean Sq F value Pr(>F)
Cond          2  0.066 0.03301   0.515  0.598
Residuals   217 13.911 0.06411

> out1 <- LSD.test(reg1, "Cond", p.adj = "none")
> out1

$`statistics`
     MSerror  Df      Mean       CV
  0.06410718 217 0.9318182 27.17203

$parameters
        test p.ajusted name.t ntr alpha
  Fisher-LSD      none   Cond   3  0.05

$means
  as.integer(SeenOther)       std  r       LCL       UCL Min Max Q25 Q50 Q75
1             0.9571429 0.2039973 70 0.8974969 1.0167889   0   1   1   1   1
2             0.9189189 0.2748228 74 0.8609074 0.9769305   0   1   1   1   1
3             0.9210526 0.2714484 76 0.8638095 0.9782958   0   1   1   1   1

$comparison
NULL

$groups
  as.integer(SeenOther) groups
1             0.9571429      a
3             0.9210526      a
```

© 2019 Chang JH et al. *JAMA Network Open.*

```
2               0.9189189      a

attr(,"class")
[1] "group"
```

Familiar with game

```
> reg1 <- aov(Familiar ~ Cond, data = ggr)
> summary(reg1)

             Df Sum Sq Mean Sq F value Pr(>F)
Cond          2    0.3  0.1311   0.079  0.924
Residuals   217  362.2  1.6690

> out1 <- LSD.test(reg1, "Cond", p.adj = "none")
> out1

$`statistics`
   MSerror  Df     Mean      CV
  1.669005 217 2.872727 44.9712

$parameters
        test p.ajusted name.t ntr alpha
  Fisher-LSD      none   Cond   3  0.05

$means
  as.numeric(Familiar)      std  r      LCL      UCL Min Max Q25 Q50 Q75
1             2.900000 1.405476 70 2.595662 3.204338   0   4   2 3.5   4
2             2.824324 1.317512 74 2.528326 3.120323   0   4   2 3.0   4
3             2.894737 1.149828 76 2.602659 3.186815   0   4   2 3.0   4

$comparison
NULL

$groups
  as.numeric(Familiar) groups
1             2.900000      a
3             2.894737      a
2             2.824324      a

attr(,"class")
[1] "group"
```

© 2019 Chang JH et al. *JAMA Network Open.*

```
> std.error(ggr$Familiar[ggr$Cond == 1])
[1] 0.1679865
> std.error(ggr$Familiar[ggr$Cond == 2])
[1] 0.1531577
> std.error(ggr$Familiar[ggr$Cond == 3])
[1] 0.1318944
```

Liked the game

```
> reg1 <- aov(LikeMV ~ Cond, data = ggr)
> summary(reg1)

             Df Sum Sq Mean Sq F value Pr(>F)
Cond          2    2.6   1.320   0.856  0.426
Residuals   217  334.9   1.543

> out1 <- LSD.test(reg1, "Cond", p.adj = "none")
> out1

$`statistics`
   MSerror  Df     Mean       CV
  1.543256 217 2.581818 48.11643

$parameters
        test p.ajusted name.t ntr alpha
  Fisher-LSD      none   Cond   3  0.05

$means
  as.numeric(LikeMV)      std  r      LCL      UCL Min Max Q25 Q50  Q75
1           2.728571 1.317897 70 2.435922 3.021220   0   4   2   3 4.00
2           2.567568 1.135734 74 2.282938 2.852197   0   4   2   3 3.75
3           2.460526 1.269549 76 2.179667 2.741386   0   4   2   3 3.25

$comparison
NULL

$groups
  as.numeric(LikeMV) groups
1           2.728571      a
2           2.567568      a
3           2.460526      a
```

© 2019 Chang JH et al. *JAMA Network Open.*

```
attr(,"class")
[1] "group"

> std.error(ggr$LikeMV[ggr$Cond == 1])
[1] 0.1575188
> std.error(ggr$LikeMV[ggr$Cond == 2])
[1] 0.1320264
> std.error(ggr$LikeMV[ggr$Cond == 3])
[1] 0.1456272
```

Game was exciting

```
> reg1 <- aov(ExciteMV ~ Cond, data = ggr)
> summary(reg1)

             Df Sum Sq Mean Sq F value Pr(>F)
Cond          2    0.6  0.2895   0.197  0.822
Residuals   217  319.3  1.4716

> out1 <- LSD.test(reg1, "Cond", p.adj = "none")
> out1

$`statistics`
   MSerror  Df     Mean       CV
  1.471567 217 2.136364 56.78255

$parameters
        test p.ajusted name.t ntr alpha
  Fisher-LSD      none   Cond   3  0.05

$means
  as.numeric(ExciteMV)      std  r      LCL      UCL Min Max Q25 Q50 Q75
1             2.171429 1.250755 70 1.885658 2.457200   0   4   1   2   3
2             2.175676 1.089905 74 1.897736 2.453616   0   4   1   2   3
3             2.065789 1.289295 76 1.791531 2.340048   0   4   1   2   3

$comparison
NULL

$groups
  as.numeric(ExciteMV) groups
```

© 2019 Chang JH et al. *JAMA Network Open.*

```
2              2.175676      a
1              2.171429      a
3              2.065789      a

attr(,"class")
[1] "group"

> std.error(ggr$ExciteMV[ggr$Cond == 1])
[1] 0.1494939
> std.error(ggr$ExciteMV[ggr$Cond == 2])
[1] 0.1266989
> std.error(ggr$ExciteMV[ggr$Cond == 3])
[1] 0.1478922
```

Game was boring

```
> reg1 <- aov(BoringMV ~ Cond, data = ggr)
> summary(reg1)

             Df Sum Sq Mean Sq F value Pr(>F)
Cond          2   1.69  0.8462   0.669  0.513
Residuals   217 274.49  1.2649

> out1 <- LSD.test(reg1, "Cond", p.adj = "none")
> out1

$`statistics`
   MSerror  Df     Mean       CV
  1.264929 217 2.909091 38.66123

$parameters
        test p.ajusted name.t ntr alpha
  Fisher-LSD      none   Cond   3  0.05

$means
  as.numeric(BoringMV)       std  r      LCL      UCL Min Max  Q25 Q50 Q75
1              2.957143 1.1090630 70 2.692195 3.222091   0   4 2.00   3   4
2              2.986486 0.9579265 74 2.728799 3.244174   0   4 2.25   3   4
3              2.789474 1.2787055 76 2.535199 3.043748   0   4 2.00   3   4

$comparison
NULL
```

© 2019 Chang JH et al. *JAMA Network Open.*

```
$groups
  as.numeric(BoringMV) groups
2             2.986486      a
1             2.957143      a
3             2.789474      a

attr(,"class")
[1] "group"

> std.error(ggr$BoringMV[ggr$Cond == 1])
[1] 0.1325584
> std.error(ggr$BoringMV[ggr$Cond == 2])
[1] 0.1113567
> std.error(ggr$BoringMV[ggr$Cond == 3])
[1] 0.1466776
```

Game was fun

```
> reg1 <- aov(FunMV ~ Cond, data = ggr)
> summary(reg1)

             Df Sum Sq Mean Sq F value Pr(>F)
Cond          2    1.4  0.7194   0.454  0.636
Residuals   217  343.9  1.5849

> out1 <- LSD.test(reg1, "Cond", p.adj = "none")
> out1

$`statistics`
   MSerror  Df     Mean       CV
  1.584886 217 2.413636 52.15875

$parameters
        test p.ajusted name.t ntr alpha
  Fisher-LSD      none   Cond   3  0.05

$means
  as.numeric(FunMV)      std  r      LCL      UCL Min Max Q25 Q50 Q75
1          2.514286 1.315932 70 2.217716 2.810856   0   4   2   3   4
2          2.418919 1.182136 74 2.130476 2.707362   0   4   1   3   3
3          2.315789 1.277607 76 2.031167 2.600412   0   4   1   2   3
```

© 2019 Chang JH et al. *JAMA Network Open.*

```
$comparison
NULL

$groups
  as.numeric(FunMV) groups
1          2.514286      a
2          2.418919      a
3          2.315789      a

attr(,"class")
[1] "group"

> std.error(ggr$FunMV[ggr$Cond == 1])
[1] 0.157284
> std.error(ggr$FunMV[ggr$Cond == 2])
[1] 0.1374205
> std.error(ggr$FunMV[ggr$Cond == 3])
[1] 0.1465516
```

Part of the action

```
> reg1 <- aov(TrnsptMV ~ Cond, data = ggr)
> summary(reg1)

             Df Sum Sq Mean Sq F value Pr(>F)
Cond          2    1.0  0.5059    0.27  0.764
Residuals   217  406.3  1.8725

> out1 <- LSD.test(reg1, "Cond", p.adj = "none")
> out1

$`statistics`
   MSerror  Df     Mean       CV
  1.872505 217 1.854545 73.78601

$parameters
        test p.ajusted name.t ntr alpha
  Fisher-LSD      none   Cond   3  0.05

$means
  as.numeric(TrnsptMV)      std   r      LCL      UCL Min Max Q25 Q50 Q75
```

© 2019 Chang JH et al. *JAMA Network Open.*

```
1              1.942857 1.295317 70 1.620498 2.265216   0   4   1   2   3
2              1.851351 1.310468 74 1.537826 2.164877   0   4   1   2   3
3              1.776316 1.484127 76 1.466943 2.085688   0   4   0   2   3

$comparison
NULL

$groups
  as.numeric(TrnsptMV) groups
1              1.942857      a
2              1.851351      a
3              1.776316      a

attr(,"class")
[1] "group"

> std.error(ggr$TrnsptMV[ggr$Cond == 1])
[1] 0.15482
> std.error(ggr$TrnsptMV[ggr$Cond == 2])
[1] 0.1523388
> std.error(ggr$TrnsptMV[ggr$Cond == 3])
[1] 0.170241
```

Game was violent

```
> reg1 <- aov(ViolMV ~ Cond, data = ggr)
> summary(reg1)

             Df  Sum Sq  Mean Sq  F value    Pr(>F)
Cond          2    40.7    20.35    18.18 5.04e-08 ***
Residuals   217   243.0     1.12
---
Signif. codes:  0 ‘***’ 0.001 ‘**’ 0.01 ‘*’ 0.05 ‘.’ 0.1 ‘ ’ 1

> out1 <- LSD.test(reg1, "Cond", p.adj = "none")
> out1

$`statistics`
   MSerror  Df     Mean       CV
  1.119739 217 1.077273 98.22743

$parameters
```

© 2019 Chang JH et al. *JAMA Network Open.*

```
        test p.ajusted name.t ntr alpha
  Fisher-LSD      none   Cond   3  0.05

$means
  as.numeric(ViolMV)       std  r        LCL       UCL Min Max Q25 Q50 Q75
1          0.4714286 0.8465005 70 0.2221492 0.720708   0   4   0   0   1
2          1.2162162 1.1499883 74 0.9737677 1.458665   0   4   0   1   2
3          1.5000000 1.1372481 76 1.2607628 1.739237   0   4   1   1   2

$comparison
NULL

$groups
  as.numeric(ViolMV) groups
3          1.5000000      a
2          1.2162162      a
1          0.4714286      b

attr(,"class")
[1] "group"

> std.error(ggr$ViolMV[ggr$Cond == 1])
[1] 0.1011762
> std.error(ggr$ViolMV[ggr$Cond == 2])
[1] 0.1336834
> std.error(ggr$ViolMV[ggr$Cond == 3])
[1] 0.1304513
```

I want to play more

```
> reg1 <- aov(SeeMV ~ Cond, data = ggr)
> summary(reg1)

             Df Sum Sq Mean Sq F value Pr(>F)
Cond          2    4.0   2.013   0.934  0.394
Residuals   217  467.4   2.154

> out1 <- LSD.test(reg1, "Cond", p.adj = "none")
> out1

$`statistics`
   MSerror  Df     Mean       CV
```

© 2019 Chang JH et al. *JAMA Network Open.*

```
  2.153717 217 2.290909 64.05993

$parameters
        test p.ajusted name.t ntr alpha
  Fisher-LSD      none   Cond   3  0.05

$means
  as.numeric(SeeMV)      std  r      LCL      UCL Min Max Q25 Q50 Q75
1          2.485714 1.471890 70 2.139996 2.831433   0   4   1 3.0   4
2          2.229730 1.521961 74 1.893485 2.565974   0   4   1 2.5   4
3          2.171053 1.408433 76 1.839262 2.502844   0   4   1 2.0   3

$comparison
NULL

$groups
  as.numeric(SeeMV) groups
1          2.485714      a
2          2.229730      a
3          2.171053      a

attr(,"class")
[1] "group"

> std.error(ggr$SeeMV[ggr$Cond == 1])
[1] 0.1759245
> std.error(ggr$SeeMV[ggr$Cond == 2])
[1] 0.1769243
> std.error(ggr$SeeMV[ggr$Cond == 3])
[1] 0.1615584
```

A friend would want to play more

```
> reg1 <- aov(FrndSeeMV ~ Cond, data = ggr)
> summary(reg1)

             Df Sum Sq Mean Sq F value Pr(>F)
Cond          2    3.4   1.694   0.952  0.388
Residuals   216  384.3   1.779
1 observation deleted due to missingness

> out1 <- LSD.test(reg1, "Cond", p.adj = "none")
```

© 2019 Chang JH et al. *JAMA Network Open.*

```
> out1

$`statistics`
   MSerror  Df     Mean       CV
  1.779258 216 2.296804 58.07586

$parameters
        test p.ajusted name.t ntr alpha
  Fisher-LSD      none   Cond   3  0.05

$means
  as.numeric(FrndSeeMV)      std  r      LCL      UCL Min Max Q25 Q50 Q75
1              2.142857 1.375623 70 1.828619 2.457095   0   4   1 2.0   3
2              2.287671 1.358916 73 1.979958 2.595385   0   4   1 2.0   3
3              2.447368 1.269065 76 2.145790 2.748947   0   4   2 2.5   4

$comparison
NULL

$groups
  as.numeric(FrndSeeMV) groups
3              2.447368      a
2              2.287671      a
1              2.142857      a

attr(,"class")
[1] "group"

> std.error(ggr$FrndSeeMV[ggr$Cond == 1])
[1] 0.1644184
> std.error(ggr$FrndSeeMV[ggr$Cond == 2])
[1] 0.159049
> std.error(ggr$FrndSeeMV[ggr$Cond == 3])
[1] 0.1455718
```

Chests opened

```
> reg1 <- aov(Chests ~ Cond, data = ggr)
> summary(reg1)

             Df Sum Sq Mean Sq F value   Pr(>F)
Cond          2   3913  1956.5      14 4.06e-06 ***
```

© 2019 Chang JH et al. *JAMA Network Open.*

```
Residuals   105  14669   139.7
---
Signif. codes:  0 ‘***’ 0.001 ‘**’ 0.01 ‘*’ 0.05 ‘.’ 0.1 ‘ ’ 1
112 observations deleted due to missingness

> out1 <- LSD.test(reg1, "Cond", p.adj = "none")
> out1

$`statistics`
   MSerror  Df     Mean       CV
  139.7075 105 20.91667 56.50897

$parameters
        test p.ajusted name.t ntr alpha
  Fisher-LSD      none   Cond   3  0.05

$means
  as.numeric(Chests)       std  r      LCL      UCL Min Max   Q25  Q50 Q75
1           29.70588 14.712455 34 25.68656 33.72520   2  71 20.00 30.0  38
2           17.94444 10.878404 36 14.03837 21.85052   0  39 11.75 17.0  24
3           15.86842  9.563927 38 12.06652 19.67032   1  32  7.00 17.5  24

$comparison
NULL

$groups
  as.numeric(Chests) groups
1           29.70588      a
2           17.94444      b
3           15.86842      b

attr(,"class")
[1] "group"

> std.error(na.omit(ggr$Chests[ggr$Cond == 1]))
[1] 2.523165
> std.error(na.omit(ggr$Chests[ggr$Cond == 2]))
[1] 1.813067
> std.error(na.omit(ggr$Chests[ggr$Cond == 3]))
[1] 1.551474
```

© 2019 Chang JH et al. *JAMA Network Open.*

Monsters killed

```
> reg1 <- aov(Kills ~ Cond, data = ggr)
> summary(reg1)

              Df Sum Sq Mean Sq F value Pr(>F)
Cond           1    3.7   3.676   0.164  0.687
Residuals     72 1612.4  22.395
146 observations deleted due to missingness

> out1 <- LSD.test(reg1, "Cond", p.adj = "none")
> out1

$`statistics`
   MSerror Df     Mean       CV
  22.39508 72 4.743243 99.77022

$parameters
        test p.ajusted name.t ntr alpha
  Fisher-LSD      none   Cond   2  0.05

$means
  as.numeric(Kills)      std  r      LCL      UCL Min Max  Q25 Q50  Q75
2          4.972222 4.668962 36 3.399930 6.544515   0  19 1.00   4 6.25
3          4.526316 4.791529 38 2.995959 6.056673   0  23 1.25   3 6.75

$comparison
NULL

$groups
  as.numeric(Kills) groups
2          4.972222      a
3          4.526316      a

attr(,"class")
[1] "group"

t.test(ggr2$Kills[ggr2$Cond == 2], ggr2$Kills[ggr2$Cond == 3])

  Welch Two Sample t-test
```

© 2019 Chang JH et al. *JAMA Network Open.*

```
data:  ggr2$Kills[ggr2$Cond == 2] and ggr2$Kills[ggr2$Cond == 3]
t = 0.40542, df = 71.94, p-value = 0.6864
alternative hypothesis: true difference in means is not equal to 0
95 percent confidence interval:
 -1.746673  2.638486
sample estimates:
mean of x mean of y
 4.972222  4.526316


> std.error(na.omit(ggr$Kills[ggr$Cond == 2]))
[1] 0.7781603
> std.error(na.omit(ggr$Kills[ggr$Cond == 3]))
[1] 0.7772886
```

Table 3

Trigger pulls

```
> reg1 <- aov(GunPull ~ Cond, data = ggr)
> summary(reg1)

             Df Sum Sq Mean Sq F value Pr(>F)
Cond          2   1250   625.0   3.624 0.0283 *
Residuals   217  37424   172.5
---
Signif. codes:  0 ‘***’ 0.001 ‘**’ 0.01 ‘*’ 0.05 ‘.’ 0.1 ‘ ’ 1

> out1 <- LSD.test(reg1, "Cond", p.adj = "none")
> out1

$`statistics`
  MSerror  Df     Mean       CV
  172.463 217 4.390909 299.0842

$parameters
        test p.ajusted name.t ntr alpha
  Fisher-LSD      none   Cond   3  0.05

$means
   GunPull       std   r        LCL       UCL Min Max Q25 Q50 Q75
```

© 2019 Chang JH et al. *JAMA Network Open.*

```
1 2.142857  8.313226 70 -0.9508266  5.236541   0  50   0   0   0
2 3.202703  9.731997 74  0.1937936  6.211612   0  50   0   0   0
3 7.618421 18.526354 76  4.6493668 10.587475   0  87   0   0   0

$comparison
NULL

$groups
   GunPull groups
3 7.618421      a
2 3.202703      b
1 2.142857      b

attr(,"class")
[1] "group"

> std.error(ggr$GunPull[ggr$Cond == 1])
[1] 0.9936206
> std.error(ggr$GunPull[ggr$Cond == 2])
[1] 1.131322
> std.error(ggr$GunPull[ggr$Cond == 3])
[1] 2.125119
```

Shooting self or partner

```
> reg1 <- aov(SelfOther ~ Cond, data = ggr)
> summary(reg1)

             Df Sum Sq Mean Sq F value Pr(>F)
Cond          2    312  155.82   3.913 0.0214 *
Residuals   217   8640   39.82
---
Signif. codes:  0 ‘***’ 0.001 ‘**’ 0.01 ‘*’ 0.05 ‘.’ 0.1 ‘ ’ 1

> out1 <- LSD.test(reg1, "Cond", p.adj = "none")
> out1

$`statistics`
   MSerror  Df     Mean       CV
  39.81658 217 1.572727 401.2163
```

© 2019 Chang JH et al. *JAMA Network Open.*

```
$parameters
        test p.ajusted name.t ntr alpha
  Fisher-LSD      none   Cond   3  0.05

$means
  SelfOther       std  r         LCL      UCL Min Max Q25 Q50 Q75
1 0.1428571 0.9054699 70 -1.34362595 1.629340   0   7   0   0   0
2 1.4054054 5.7000815 74 -0.04034437 2.851155   0  43   0   0   0
3 3.0526316 9.1007615 76  1.62603165 4.479232   0  61   0   0   0

$comparison
NULL

$groups
  SelfOther groups
3 3.0526316      a
2 1.4054054     ab
1 0.1428571      b

attr(,"class")
[1] "group"

> std.error(ggr$SelfOther[ggr$Cond == 1])
[1] 0.1082244
> std.error(ggr$SelfOther[ggr$Cond == 2])
[1] 0.662621
> std.error(ggr$SelfOther[ggr$Cond == 3])
[1] 1.043929
```

Touched gun

```
> table(ggr$TouchGun, ggr$Cond)

     1  2  3
  0 39 32 29
  1 31 42 47


> reg1 <- aov(TouchGun ~ Cond, data = ggr)
> summary(reg1)
```

© 2019 Chang JH et al. *JAMA Network Open.*

```
  Df Sum Sq Mean Sq F value Pr(>F)
Cond          2   1.18  0.5888   2.394 0.0936 .
Residuals   217  53.37  0.2459
---
Signif. codes:  0 ‘***’ 0.001 ‘**’ 0.01 ‘*’ 0.05 ‘.’ 0.1 ‘ ’ 1

> out1 <- LSD.test(reg1, "Cond", p.adj = "none") $`statistics`
    MSerror  Df      Mean       CV
  0.2459346 217 0.5454545 90.91828

$parameters
        test p.ajusted name.t ntr alpha
  Fisher-LSD      none   Cond   3  0.05

$means
   TouchGun       std  r       LCL       UCL Min Max Q25 Q50 Q75
1 0.4428571 0.5003105 70 0.3260316 0.5596827   0   1   0   0   1
2 0.5675676 0.4987953 74 0.4539433 0.6811918   0   1   0   1   1
3 0.6184211 0.4890018 76 0.5063019 0.7305403   0   1   0   1   1

$comparison
NULL

$groups
   TouchGun groups
3 0.6184211      a
2 0.5675676     ab
1 0.4428571      b

attr(,"class")
[1] "group"
```

Time with gun

```
> reg1 <- aov(GunTime ~ Cond, data = ggr)
> summary(reg1)

             Df  Sum Sq Mean Sq F value  Pr(>F)
Cond          2   76347   38173   2.738  0.067 .
Residuals   217 3025941   13944
```

© 2019 Chang JH et al. *JAMA Network Open.*

```
---
Signif. codes:  0 ‘***’ 0.001 ‘**’ 0.01 ‘*’ 0.05 ‘.’ 0.1 ‘ ’ 1

> out1 <- LSD.test(reg1, "Cond", p.adj = "none")
> out1

$`statistics`
   MSerror  Df     Mean       CV
  13944.43 217 52.62273 224.4022

$parameters
        test p.ajusted name.t ntr alpha
  Fisher-LSD      none   Cond   3  0.05

$means
   GunTime       std  r       LCL      UCL Min Max Q25 Q50   Q75
1 25.35714  78.11994 70 -2.461013 53.17530   0 452   0 0.0  5.75
2 65.74324 153.70173 74 38.687373 92.79911   0 879   0 1.0 29.00
3 64.96053 108.33804 76 38.263028 91.65802   0 430   0 8.5 71.50

$comparison
NULL

$groups
   GunTime groups
2 65.74324      a
3 64.96053      a
1 25.35714      b

attr(,"class")
[1] "group"

> std.error(ggr$GunTime[ggr$Cond == 1])
[1] 9.337119
> std.error(ggr$GunTime[ggr$Cond == 2])
[1] 17.86746
> std.error(ggr$GunTime[ggr$Cond == 3])
[1] 12.42723
```

Methods

© 2019 Chang JH et al. *JAMA Network Open.*

Calculation of Cronbach's alpha for media diet

```
> ggr$TV1ExpS <- ggr$tv1*ggr$TV1Rating
> ggr$TV2ExpS <- ggr$tv2*ggr$TV2Rating
> ggr$TV3ExpS <- ggr$tv3*ggr$TV3Rating
>
> tvscale <- as.data.frame(cbind(ggr$TV1ExpS, ggr$TV2ExpS, ggr$TV3ExpS))
> ggr$MeanTVExp <- rowMeans(tvscale, na.rm = TRUE)
>
>
> ggr$M1ExpS <- ggr$mv1*ggr$MV1Rating
> ggr$M2ExpS <- ggr$mv2*ggr$MV2Rating
> ggr$M3ExpS <- ggr$mv3*ggr$MV3Rating
>
> mvscale <- as.data.frame(cbind(ggr$M1ExpS, ggr$M2ExpS, ggr$M3ExpS))
> ggr$MeanMVExp <- rowMeans(mvscale, na.rm = TRUE)
>
>
> ggr$VG2Rating <- as.numeric(ggr$VG2Rating)
> ggr$VG1ExpS <- ggr$vg1*ggr$VG1Rating
> ggr$VG2ExpS <- ggr$vg2*ggr$VG2Rating
> ggr$VG3ExpS <- ggr$vg3*ggr$VG3Rating
>
> vgscale <- as.data.frame(cbind(ggr$VG1ExpS, ggr$VG2ExpS, ggr$VG3ExpS))
> ggr$MeanVGExp <- rowMeans(vgscale, na.rm = TRUE)
>
> media <- as.data.frame(cbind(tvscale, mvscale, mvscale))
> names <- c("v1", "v2", "v3", "v4", "v5", "v6", "v7", "v8", "v9")
> names(media) <- names

> alpha(media, na.rm = TRUE)

Reliability analysis
Call: alpha(x = media)

  raw_alpha std.alpha G6(smc) average_r S/N   ase mean sd median_r
      0.73      0.76    0.92      0.26 3.1 0.029  5.9  2     0.15

 lower alpha upper     95% confidence boundaries
```

© 2019 Chang JH et al. *JAMA Network Open.*

```
0.67 0.73 0.78

 Reliability if an item is dropped:
   raw_alpha std.alpha G6(smc) average_r S/N alpha se var.r med.r
v1      0.72      0.75    0.93      0.28 3.0    0.030 0.074  0.16
v2      0.74      0.76    0.94      0.29 3.2    0.028 0.076  0.16
v3      0.71      0.75    0.92      0.27 3.0    0.031 0.074  0.16
v4      0.69      0.72    0.86      0.24 2.5    0.032 0.058  0.15
v5      0.70      0.72    0.86      0.24 2.6    0.032 0.058  0.14
v6      0.70      0.73    0.86      0.25 2.7    0.031 0.059  0.15
v7      0.69      0.72    0.86      0.24 2.5    0.032 0.058  0.15
v8      0.70      0.72    0.86      0.24 2.6    0.032 0.058  0.14
v9      0.70      0.73    0.86      0.25 2.7    0.031 0.059  0.15

 Item statistics
     n raw.r std.r r.cor r.drop mean  sd
v1 217  0.55  0.48  0.36   0.35  7.4 4.3
v2 214  0.52  0.43  0.27   0.28  6.9 4.7
v3 211  0.57  0.49  0.38   0.38  7.3 4.3
v4 219  0.62  0.67  0.70   0.49  5.8 3.1
v5 218  0.59  0.65  0.68   0.47  5.0 2.8
v6 214  0.57  0.60  0.63   0.43  5.0 3.1
v7 219  0.62  0.67  0.70   0.49  5.8 3.1
v8 218  0.59  0.65  0.68   0.47  5.0 2.8
v9 214  0.57  0.60  0.63   0.43  5.0 3.1
```

Cronbach's alpha of trait aggression

```
> behavior <- as.data.frame(cbind(ggr$Beh1, ggr$Beh2, ggr$Beh3, ggr$Beh4, ggr$Beh5, ggr$Beh6, ggr$Beh
7, ggr$Beh8, ggr$Beh9))
> alpha(behavior, na.rm = TRUE)

Reliability analysis
Call: alpha(x = behavior)

  raw_alpha std.alpha G6(smc) average_r S/N   ase mean   sd median_r
      0.76      0.77    0.82      0.27 3.3 0.024 0.74 0.46     0.26

 lower alpha upper     95% confidence boundaries
0.71 0.76 0.81

 Reliability if an item is dropped:
```

© 2019 Chang JH et al. *JAMA Network Open.*

```
   raw_alpha std.alpha G6(smc) average_r S/N alpha se var.r med.r
V1      0.73      0.75    0.75      0.27 3.0    0.028 0.021  0.29
V2      0.76      0.77    0.77      0.30 3.4    0.024 0.013  0.29
V3      0.75      0.76    0.81      0.28 3.1    0.026 0.035  0.28
V4      0.73      0.74    0.80      0.26 2.8    0.028 0.036  0.24
V5      0.74      0.75    0.80      0.27 2.9    0.027 0.034  0.26
V6      0.73      0.73    0.79      0.26 2.8    0.028 0.031  0.24
V7      0.75      0.76    0.81      0.29 3.2    0.025 0.030  0.29
V8      0.73      0.73    0.79      0.26 2.8    0.027 0.029  0.24
V9      0.73      0.73    0.78      0.26 2.8    0.027 0.028  0.25

 Item statistics
     n raw.r std.r r.cor r.drop mean   sd
V1  78  0.63  0.59  0.61   0.52 1.31 0.96
V2 220  0.43  0.44  0.43   0.32 1.29 0.98
V3 220  0.54  0.54  0.44   0.38 0.61 0.75
V4 220  0.68  0.65  0.57   0.52 1.29 1.01
V5 220  0.59  0.61  0.52   0.45 0.39 0.64
V6 220  0.67  0.67  0.60   0.52 0.67 0.78
V7 220  0.48  0.51  0.41   0.33 0.31 0.52
V8 220  0.65  0.67  0.61   0.50 0.49 0.67
V9 215  0.67  0.67  0.63   0.50 0.66 0.80

Non missing response frequency for each item
      0    1    3 miss
V1 0.13 0.65 0.22 0.65
V2 0.15 0.62 0.22 0.00
V3 0.50 0.45 0.05 0.00
V4 0.17 0.60 0.23 0.00
V5 0.66 0.31 0.03 0.00
V6 0.46 0.48 0.06 0.00
V7 0.70 0.29 0.01 0.00
V8 0.58 0.39 0.03 0.00
V9 0.48 0.45 0.07 0.02
```

Cronbach's alpha of opinion toward guns

```
> gunopinion <- as.data.frame(cbind(ggr$GunQ1, ggr$GunQ2, ggr$GunQ3, ggr$GunQ4, ggr$GunQ5, ggr$GunQ6,
ggr$GunQ7, ggr$GunQ8, ggr$GunQ9, ggr$GunQ10, ggr$GunQ11, ggr$GunQ12, ggr$GunQ13, ggr$GunQ14, ggr$GunQ
15))
> alpha(gunopinion, na.rm =TRUE)
```

© 2019 Chang JH et al. *JAMA Network Open.*

```
Reliability analysis
Call: alpha(x = gunopinion, na.rm = TRUE)

  raw_alpha std.alpha G6(smc) average_r S/N   ase mean   sd median_r
      0.85      0.86    0.89      0.28 5.9 0.015    3 0.61     0.24

 lower alpha upper     95% confidence boundaries
0.82 0.85 0.87

 Reliability if an item is dropped:
    raw_alpha std.alpha G6(smc) average_r S/N alpha se var.r med.r
V1       0.83      0.84    0.88      0.28 5.4    0.016 0.024  0.23
V2       0.83      0.84    0.88      0.28 5.4    0.016 0.022  0.23
V3       0.83      0.84    0.88      0.28 5.4    0.016 0.023  0.24
V4       0.82      0.84    0.87      0.27 5.2    0.017 0.022  0.23
V5       0.84      0.85    0.88      0.28 5.5    0.016 0.023  0.24
V6       0.83      0.84    0.88      0.28 5.4    0.016 0.023  0.23
V7       0.83      0.84    0.88      0.27 5.3    0.017 0.024  0.23
V8       0.83      0.84    0.88      0.28 5.3    0.016 0.024  0.23
V9       0.84      0.84    0.88      0.28 5.4    0.016 0.023  0.24
V10      0.84      0.85    0.88      0.28 5.5    0.016 0.023  0.23
V11      0.84      0.85    0.88      0.28 5.6    0.016 0.023  0.24
V12      0.83      0.85    0.88      0.28 5.5    0.016 0.025  0.23
V13      0.84      0.85    0.88      0.29 5.7    0.016 0.026  0.26
V14      0.85      0.86    0.89      0.31 6.2    0.014 0.021  0.28
V15      0.85      0.86    0.88      0.30 5.9    0.015 0.023  0.28

 Item statistics
      n raw.r std.r r.cor r.drop mean   sd
V1  216  0.62  0.63  0.59   0.54  3.2 1.03
V2  216  0.63  0.62  0.60   0.56  3.1 1.09
V3  215  0.63  0.60  0.58   0.53  2.9 1.30
V4  217  0.72  0.70  0.69   0.65  2.5 1.31
V5  214  0.57  0.58  0.54   0.50  3.2 0.92
V6  215  0.62  0.61  0.58   0.54  3.0 1.06
V7  216  0.67  0.67  0.65   0.58  3.1 1.26
V8  216  0.63  0.65  0.62   0.55  3.2 1.08
V9  215  0.56  0.61  0.59   0.49  3.6 0.69
V10 215  0.53  0.59  0.56   0.48  3.7 0.63
V11 215  0.49  0.55  0.52   0.42  3.7 0.71
V12 216  0.59  0.58  0.54   0.50  2.8 1.14
```

© 2019 Chang JH et al. *JAMA Network Open.*

```
v13 214  0.52  0.50  0.44   0.42  2.2 1.19
v14 217  0.34  0.32  0.25   0.21  2.5 1.28
v15 215  0.46  0.41  0.36   0.33  2.2 1.27

Non missing response frequency for each item
       0    1    2    3    4 miss
v1  0.03 0.04 0.17 0.23 0.53 0.02
v2  0.04 0.04 0.18 0.26 0.48 0.02
v3  0.08 0.07 0.20 0.19 0.46 0.02
v4  0.08 0.17 0.26 0.17 0.32 0.01
v5  0.01 0.05 0.14 0.33 0.47 0.03
v6  0.02 0.09 0.19 0.29 0.41 0.02
v7  0.06 0.11 0.10 0.18 0.56 0.02
v8  0.04 0.05 0.12 0.27 0.52 0.02
v9  0.00 0.02 0.03 0.25 0.70 0.02
v10 0.00 0.01 0.05 0.12 0.82 0.02
v11 0.01 0.01 0.04 0.19 0.75 0.02
v12 0.02 0.12 0.31 0.16 0.38 0.02
v13 0.07 0.17 0.43 0.10 0.22 0.03
v14 0.06 0.17 0.29 0.13 0.35 0.01
v15 0.08 0.23 0.34 0.09 0.26 0.02
```

Results

Random Assignment Check

Finding handgun

```
> table(gg.trim$FoundGun, gg.trim$Cond)

     1  2  3
  0  8  8  6
  1 70 74 76

> fisher.test(gg.trim$FoundGun, gg.trim$Cond)

  Fisher's Exact Test for Count Data

data:  gg.trim$FoundGun and gg.trim$Cond
```

© 2019 Chang JH et al. *JAMA Network Open.*

```
p-value = 0.8506
alternative hypothesis: two.sided

> table(ggr$TouchGun, ggr$ToldAbtGun)

     0  1
  0 87 13
  1 85 35

> table(ggr$TouchGun, ggr$Cond)

     1  2  3
  0 39 32 29
  1 31 42 47

> table(ggr$OnePull)

  0   1
181  39

> sum(ggr$GunPull)
[1] 966
> sum(ggr$SelfOther)
[1] 346
```

Touched handgun

```
> table1 <- table(ggr$Cond, ggr$TouchGun)

> table1

     0  1
  1 39 31
  2 32 42
  3 29 47

> chisq.post.hoc(table1)
Adjusted p-values used the fdr method.
```

© 2019 Chang JH et al. *JAMA Network Open.*

```
  comparison  raw.p  adj.p
1    1 vs. 2 0.1820 0.2730
2    1 vs. 3 0.0461 0.1382
3    2 vs. 3 0.6183 0.6183

> chisq.test(table1)

  Pearson's Chi-squared test

data:  table1
X-squared = 4.7499, df = 2, p-value = 0.09302


> fisher.test(ggr$Cond, ggr$TouchGun)

  Fisher's Exact Test for Count Data

data:  ggr$Cond and ggr$TouchGun
p-value = 0.09477
alternative hypothesis: two.sided

> chisq.test(table(ggr$Cond[ggr$Cond != 2], ggr$TouchGun[ggr$Cond !=2]))

  Pearson's Chi-squared test with Yates' continuity correction

data:  table(ggr$Cond[ggr$Cond != 2], ggr$TouchGun[ggr$Cond != 2])
X-squared = 3.8357, df = 1, p-value = 0.05017


> mean(na.omit(ggr2$GunTime[ggr2$TouchGun == 1]))
[1] 96.475
> sd(na.omit(ggr2$GunTime[ggr2$TouchGun == 1]))
[1] 147.6599
```

Models

```
. log using "Z:\BAC_0658_Bushman\programs\Games_and_Guns_trim_non_finders", replace
--------------------------------------------------------------------------------------------------------------------
```

© 2019 Chang JH et al. *JAMA Network Open.*

```
      name:  <unnamed>
       log:  Z:\BAC_0658_Bushman\programs\Games_and_Guns_trim_non_finders.smcl
  log type:  smcl
 opened on:  27 Mar 2019, 10:32:00

.
.
.
. use "Z:\BAC_0658_Bushman\data\Games_and_Guns_trim_non_finders_removed.dta"

.
.
.
. recode NumGuns 0=0 1/6=1, gen(AnyGunsInHouse)
(52 differences between NumGuns and AnyGunsInHouse)

.
. recode Cond 2=1 1=0 3=0, gen(Condition_Sword)
(220 differences between Cond and Condition_Sword)

.
. recode Cond 3=1 1=0 2=0, gen(Condition_Gun)
(220 differences between Cond and Condition_Gun)

.
.
.
. * Full Models

.
. nbreg GunPull Condition_Gun Condition_Sword Sex GunQMn BehMean MediaExpS GunInt Age AnyGunsInHouse GunSafety, clu
> ster(Pair)

Fitting Poisson model:
```

© 2019 Chang JH et al. *JAMA Network Open.*

```
Iteration 0:   log pseudolikelihood = -1256.9706
Iteration 1:   log pseudolikelihood = -1256.7198
Iteration 2:   log pseudolikelihood = -1256.7198

Fitting constant-only model:

Iteration 0:   log pseudolikelihood = -502.14571
Iteration 1:   log pseudolikelihood = -238.09343
Iteration 2:   log pseudolikelihood = -236.81481
Iteration 3:   log pseudolikelihood = -236.81448
Iteration 4:   log pseudolikelihood = -236.81448

Fitting full model:

Iteration 0:   log pseudolikelihood =  -232.8013
Iteration 1:   log pseudolikelihood = -225.99382
Iteration 2:   log pseudolikelihood = -225.42039
Iteration 3:   log pseudolikelihood = -225.41267
Iteration 4:   log pseudolikelihood = -225.41266

Negative binomial regression                    Number of obs     =        205
                                                Wald chi2(10)     =      67.72
Dispersion          = mean                      Prob > chi2       =     0.0000
Log pseudolikelihood = -225.41266               Pseudo R2         =     0.0481

                                 (Std. Err. adjusted for 108 clusters in Pair)
------------------------------------------------------------------------------
                |               Robust
        GunPull |      Coef.   Std. Err.      z    P>|z|     [95% Conf. Interval]
----------------+-------------------------------------------------------------
  Condition_Gun |   .8567005   .9007843     0.95   0.342    -.9088042    2.622205
Condition_Sword |   .8979552   .9807646     0.92   0.360    -1.024308    2.820218
            Sex |  -.4323485   .6143092    -0.70   0.482    -1.636372    .7716754
```

© 2019 Chang JH et al. *JAMA Network Open.*

```
    GunQMn      | -1.316456  .5194309   -2.53  0.011   -2.334522  -.2983903
   BehMean      |  2.604139  .7455959    3.49  0.000    1.142798    4.06548
  MediaExpS     |  .3396133  .1744809    1.95  0.052   -.002363    .6815896
    GunInt      |  1.039165  .2337897    4.44  0.000    .5809454   1.497384
      Age       | -.2225586  .1589667   -1.40  0.162   -.5341277   .0890104
AnyGunsInHouse  | -.1730327  .9157799   -0.19  0.850   -1.967928   1.621863
   GunSafety    | -1.891773  .8514474   -2.22  0.026   -3.560579  -.2229667
     _cons      |  .5042148  2.064174    0.24  0.807   -3.541491   4.549921
----------------+-------------------------------------------------------------
    /lnalpha    |  2.798281  .2356222                    2.33647   3.260092
----------------+-------------------------------------------------------------
     alpha      |   16.4164  3.868068                   10.34465   26.05193
------------------------------------------------------------------------------

.
. nbreg SelfOther Condition_Gun Condition_Sword Sex GunQMn BehMean MediaExpS GunInt Age AnyGunsInHouse GunSafety, c
> luster(Pair)

Fitting Poisson model:

Iteration 0:   log pseudolikelihood =  -518.2505
Iteration 1:   log pseudolikelihood = -513.78261
Iteration 2:   log pseudolikelihood =  -513.7114
Iteration 3:   log pseudolikelihood = -513.71138

Fitting constant-only model:

Iteration 0:   log pseudolikelihood = -339.48177
Iteration 1:   log pseudolikelihood = -151.59496
Iteration 2:   log pseudolikelihood = -151.52021
Iteration 3:   log pseudolikelihood = -151.52013
Iteration 4:   log pseudolikelihood = -151.52013

Fitting full model:
```

© 2019 Chang JH et al. *JAMA Network Open.*

Iteration 0: log pseudolikelihood = -148.16189 (not concave)
Iteration 1: log pseudolikelihood = -143.02753
Iteration 2: log pseudolikelihood = -139.04037
Iteration 3: log pseudolikelihood = -136.67917
Iteration 4: log pseudolikelihood = -136.54239
Iteration 5: log pseudolikelihood = -136.54193
Iteration 6: log pseudolikelihood = -136.54193

Negative binomial regression Number of obs = 205
Wald chi2(10) = 113.99
Dispersion = mean Prob > chi2 = 0.0000
Log pseudolikelihood = -136.54193 Pseudo R2 = 0.0989

(Std. Err. adjusted for 108 clusters in Pair)

SelfOther	Coef.	Robust Std. Err.	z	P>|z|	[95% Conf. Interval]	
Condition_Gun	1.929443	.9131453	2.11	0.035	.139711	3.719175
Condition_Sword	2.50358	.9500812	2.64	0.008	.6414548	4.365705
Sex	-.2898032	.5640914	-0.51	0.607	-1.395402	.8157958
GunQMn	-.2107497	.5234226	-0.40	0.687	-1.236639	.8151396
BehMean	3.246105	.7484483	4.34	0.000	1.779173	4.713036
MediaExpS	.6297028	.1900981	3.31	0.001	.2571174	1.002288
GunInt	1.011085	.1956327	5.17	0.000	.6276521	1.394518
Age	-.3904776	.1323407	-2.95	0.003	-.6498606	-.1310946
AnyGunsInHouse	-3.001787	1.036508	-2.90	0.004	-5.033305	-.9702687
GunSafety	1.184803	1.10168	1.08	0.282	-.974451	3.344057
_cons	-6.133177	2.237096	-2.74	0.006	-10.5178	-1.74855
/lnalpha	2.741236	.2948757			2.16329	3.319182
alpha	15.50614	4.572383			8.699714	27.63772

© 2019 Chang JH et al. *JAMA Network Open.*

```
------------------------------------------------------------------------------------

.
. nbreg GunTime Condition_Gun Condition_Sword Sex GunQMn BehMean MediaExpS GunInt Age AnyGunsInHouse GunSafety, clu
> ster(Pair)

Fitting Poisson model:

Iteration 0:   log pseudolikelihood = -10997.047
Iteration 1:   log pseudolikelihood = -10994.005
Iteration 2:   log pseudolikelihood = -10994.005

Fitting constant-only model:

Iteration 0:   log pseudolikelihood = -1001.5195
Iteration 1:   log pseudolikelihood = -671.59079
Iteration 2:   log pseudolikelihood = -671.52093
Iteration 3:   log pseudolikelihood = -671.52091

Fitting full model:

Iteration 0:   log pseudolikelihood = -662.84693
Iteration 1:   log pseudolikelihood = -656.98485
Iteration 2:   log pseudolikelihood = -655.60209
Iteration 3:   log pseudolikelihood = -655.58901
Iteration 4:   log pseudolikelihood =   -655.589

Negative binomial regression                    Number of obs     =        205
                                                Wald chi2(10)     =      57.62
Dispersion           = mean                     Prob > chi2       =     0.0000
Log pseudolikelihood =   -655.589               Pseudo R2         =     0.0237

                                  (Std. Err. adjusted for 108 clusters in Pair)
------------------------------------------------------------------------------------
```

© 2019 Chang JH et al. *JAMA Network Open.*

```
             |               Robust
     GunTime         |     Coef.   Std. Err.      z    P>|z|     [95% Conf. Interval]
----------------+----------------------------------------------------------------
 Condition_Gun       |   .9381426   .6056702     1.55   0.121    -.2489492    2.125234
Condition_Sword      |   1.096266   .6685329     1.64   0.101    -.2140339    2.406567
         Sex         |   .1163542   .3482742     0.33   0.738    -.5662506    .7989591
      GunQMn         |  -.9139013    .362132    -2.52   0.012    -1.623667   -.2041357
     BehMean         |   1.439592   .4896752     2.94   0.003     .4798466    2.399338
   MediaExpS         |   .0347457   .1076068     0.32   0.747    -.1761598    .2456511
      GunInt         |   .5017217    .153473     3.27   0.001     .2009202    .8025233
         Age         |  -.2967501   .1247082    -2.38   0.017    -.5411737   -.0523265
 AnyGunsInHouse      |   .5678246   .5243892     1.08   0.279    -.4599594    1.595609
    GunSafety        |   -2.25475   .6207003    -3.63   0.000      -3.4713   -1.038199
       _cons         |   6.365413   1.778243     3.58   0.000      2.88012    9.850705
----------------+----------------------------------------------------------------
     /lnalpha        |   1.973591   .1435721                      1.692195    2.254987
----------------+----------------------------------------------------------------
       alpha         |   7.196471   1.033213                      5.431387    9.535169
---------------------------------------------------------------------------------

.
.
.
. * Mini Models:

.
. nbreg GunPull Condition_Gun Condition_Sword Sex, cluster(Pair)

Fitting Poisson model:

Iteration 0:   log pseudolikelihood = -1955.2704
Iteration 1:   log pseudolikelihood = -1955.2647
Iteration 2:   log pseudolikelihood = -1955.2647
```

© 2019 Chang JH et al. *JAMA Network Open.*

```
Fitting constant-only model:

Iteration 0:   log pseudolikelihood = -568.83879
Iteration 1:   log pseudolikelihood = -278.75931
Iteration 2:   log pseudolikelihood = -276.15524
Iteration 3:   log pseudolikelihood = -276.15489
Iteration 4:   log pseudolikelihood = -276.15489

Fitting full model:

Iteration 0:   log pseudolikelihood = -274.48604
Iteration 1:   log pseudolikelihood = -274.24164
Iteration 2:   log pseudolikelihood = -274.22861
Iteration 3:   log pseudolikelihood = -274.22859

Negative binomial regression                    Number of obs     =        220
                                                Wald chi2(3)      =       6.08
Dispersion          = mean                      Prob > chi2       =     0.1079
Log pseudolikelihood = -274.22859               Pseudo R2         =     0.0070

                                  (Std. Err. adjusted for 110 clusters in Pair)
------------------------------------------------------------------------------
                |               Robust
        GunPull |      Coef.   Std. Err.      z    P>|z|     [95% Conf. Interval]
----------------+-------------------------------------------------------------
  Condition_Gun |   1.219752   .7346883     1.66   0.097    -.2202108    2.659714
Condition_Sword |   .1725817   .7715978     0.22   0.823    -1.339722    1.684886
            Sex |   .7092787    .458004     1.55   0.121    -.1883927     1.60695
          _cons |      .3876   .7028499     0.55   0.581    -.9899604     1.76516
----------------+-------------------------------------------------------------
       /lnalpha |   3.066934   .2272062                      2.621618     3.51225
----------------+-------------------------------------------------------------
          alpha |   21.47596   4.879471                      13.75797    33.52361
------------------------------------------------------------------------------
```

© 2019 Chang JH et al. *JAMA Network Open.*

```
.
. nbreg SelfOther Condition_Gun Condition_Sword Sex, cluster(Pair)

Fitting Poisson model:

Iteration 0:   log pseudolikelihood = -804.88059
Iteration 1:   log pseudolikelihood = -803.21969
Iteration 2:   log pseudolikelihood = -803.20183
Iteration 3:   log pseudolikelihood = -803.20183

Fitting constant-only model:

Iteration 0:   log pseudolikelihood = -378.17837
Iteration 1:   log pseudolikelihood = -172.61838
Iteration 2:   log pseudolikelihood = -172.61544
Iteration 3:   log pseudolikelihood = -172.61544

Fitting full model:

Iteration 0:   log pseudolikelihood = -169.82842
Iteration 1:   log pseudolikelihood = -168.38404
Iteration 2:   log pseudolikelihood = -168.36687
Iteration 3:   log pseudolikelihood = -168.36685
Iteration 4:   log pseudolikelihood = -168.36685

Negative binomial regression                    Number of obs     =        220
                                                Wald chi2(3)      =       8.42
Dispersion          = mean                      Prob > chi2       =     0.0380
Log pseudolikelihood = -168.36685               Pseudo R2         =     0.0246

                                   (Std. Err. adjusted for 110 clusters in Pair)
------------------------------------------------------------------------------
             |               Robust
```

© 2019 Chang JH et al. *JAMA Network Open.*

```
     SelfOther           |      Coef.   Std. Err.      z    P>|z|     [95% Conf. Interval]
----------------+----------------------------------------------------------------
 Condition_Gun      |   2.910076    1.08019     2.69   0.007     .7929424    5.027209
Condition_Sword    |   2.080395   1.120358     1.86   0.063     -.115465    4.276256
          Sex              |   .4002382   .3952875     1.01   0.311    -.3745111    1.174987
        _cons           |  -2.074133   1.039298    -2.00   0.046    -4.111119   -.0371472
----------------+----------------------------------------------------------------
      /lnalpha           |    3.23901   .2960979                      2.658669    3.819352
----------------+----------------------------------------------------------------
        alpha            |   25.50847   7.553003                      14.27728    45.57465
---------------------------------------------------------------------------------

.
. nbreg GunTime Condition_Gun Condition_Sword Sex, cluster(Pair)

Fitting Poisson model:

Iteration 0:   log pseudolikelihood = -15921.501
Iteration 1:   log pseudolikelihood = -15921.338
Iteration 2:   log pseudolikelihood = -15921.338

Fitting constant-only model:

Iteration 0:   log pseudolikelihood = -1093.9698
Iteration 1:   log pseudolikelihood = -743.63725
Iteration 2:   log pseudolikelihood = -743.58569
Iteration 3:   log pseudolikelihood = -743.58568

Fitting full model:

Iteration 0:   log pseudolikelihood =  -740.2443
Iteration 1:   log pseudolikelihood = -739.83206
Iteration 2:   log pseudolikelihood = -739.81562
Iteration 3:   log pseudolikelihood = -739.81561
```

© 2019 Chang JH et al. *JAMA Network Open.*

```
Negative binomial regression                    Number of obs     =        220
                                                Wald chi2(3)      =      10.28
Dispersion          = mean                      Prob > chi2       =     0.0164
Log pseudolikelihood = -739.81561               Pseudo R2         =     0.0051

                                  (Std. Err. adjusted for 110 clusters in Pair)
------------------------------------------------------------------------------
             |               Robust
     GunTime |      Coef.   Std. Err.      z    P>|z|     [95% Conf. Interval]
-------------+----------------------------------------------------------------
 Condition_Gun |   .9301017    .530476     1.75   0.080    -.1096122    1.969816
Condition_Sword |   .6860791   .5756471     1.19   0.233    -.4421685    1.814327
         Sex |    .773064   .3587233     2.16   0.031     .0699792    1.476149
       _cons |   2.813095   .4511453     6.24   0.000     1.928866    3.697323
-------------+----------------------------------------------------------------
    /lnalpha |   2.107779   .1402549                      1.832885    2.382674
-------------+----------------------------------------------------------------
       alpha |   8.229946    1.15429                      6.251896    10.83383
------------------------------------------------------------------------------

.
.
.
. * For percentiles of Predicted values, by Condition

.
. sort Cond

.
.
.
. nbreg GunPull Condition_Gun Condition_Sword Sex GunQMn BehMean MediaExpS GunInt Age AnyGunsInHouse GunSafety, clu
> ster(Pair)
```

© 2019 Chang JH et al. *JAMA Network Open.*

```
Fitting Poisson model:

Iteration 0:   log pseudolikelihood = -1256.9706
Iteration 1:   log pseudolikelihood = -1256.7198
Iteration 2:   log pseudolikelihood = -1256.7198

Fitting constant-only model:

Iteration 0:   log pseudolikelihood = -502.14571
Iteration 1:   log pseudolikelihood = -238.09343
Iteration 2:   log pseudolikelihood = -236.81481
Iteration 3:   log pseudolikelihood = -236.81448
Iteration 4:   log pseudolikelihood = -236.81448

Fitting full model:

Iteration 0:   log pseudolikelihood =  -232.8013
Iteration 1:   log pseudolikelihood = -225.99382
Iteration 2:   log pseudolikelihood = -225.42039
Iteration 3:   log pseudolikelihood = -225.41267
Iteration 4:   log pseudolikelihood = -225.41266

Negative binomial regression                    Number of obs     =        205
                                                Wald chi2(10)     =      67.72
Dispersion           = mean                     Prob > chi2       =     0.0000
Log pseudolikelihood = -225.41266               Pseudo R2         =     0.0481

                                  (Std. Err. adjusted for 108 clusters in Pair)
------------------------------------------------------------------------------
             |               Robust
     GunPull |      Coef.   Std. Err.      z    P>|z|     [95% Conf. Interval]
-------------+----------------------------------------------------------------
 Condition_Gun |   .8567005   .9007843     0.95   0.342    -.9088042    2.622205
```

© 2019 Chang JH et al. *JAMA Network Open.*

```
Condition_Sword  |  .8979552  .9807646   0.92  0.360  -1.024308   2.820218
          Sex    | -.4323485  .6143092  -0.70  0.482  -1.636372   .7716754
       GunQMn    | -1.316456  .5194309  -2.53  0.011  -2.334522  -.2983903
      BehMean    |  2.604139  .7455959   3.49  0.000   1.142798   4.06548
    MediaExpS    |  .3396133  .1744809   1.95  0.052   -.002363   .6815896
       GunInt    |  1.039165  .2337897   4.44  0.000   .5809454   1.497384
          Age    | -.2225586  .1589667  -1.40  0.162  -.5341277   .0890104
 AnyGunsInHouse  | -.1730327  .9157799  -0.19  0.850  -1.967928   1.621863
    GunSafety    | -1.891773  .8514474  -2.22  0.026  -3.560579  -.2229667
        _cons    |  .5042148  2.064174   0.24  0.807  -3.541491   4.549921
----------------+-------------------------------------------------------------
     /lnalpha    |  2.798281  .2356222                  2.33647   3.260092
----------------+-------------------------------------------------------------
        alpha    |   16.4164  3.868068                 10.34465   26.05193
------------------------------------------------------------------------------

.
. predict GunPull_full_Predicted, n
(15 missing values generated)

.
. by Cond: summarize GunPull_full_Predicted, detail

-------------------------------------------------------------------------------------------------------------
-> Cond = 1

             Predicted number of events
-------------------------------------------------------------
      Percentiles      Smallest
 1%     .0032399       .0032399
 5%     .0096932       .0051573
10%     .0263433       .0073157       Obs                69
25%     .0850878       .0096932       Sum of Wgt.        69
```

© 2019 Chang JH et al. *JAMA Network Open.*

```
50%     .3814798                    Mean          4.979842
                 Largest       Std. Dev.      16.55556
75%     2.019599       29.10579
90%     4.859893       47.56834       Variance       274.0864
95%     29.10579       82.95983       Skewness       4.551195
99%     99.00909       99.00909       Kurtosis       23.6044

--------------------------------------------------------------------------------------------------------------------
-> Cond = 2

             Predicted number of events
-------------------------------------------------------------
     Percentiles      Smallest
 1%     .0163895       .0163895
 5%     .0268972       .0171086
10%     .0559954       .0248042       Obs                66
25%     .2824344       .0268972       Sum of Wgt.         66

50%     1.414626                    Mean          96.95142
                 Largest       Std. Dev.      577.6941
75%     8.781828       198.2073
90%     52.15439       447.0967       Variance       333730.5
95%     198.2073       670.8679       Skewness       7.595813
99%     4648.285       4648.285       Kurtosis       60.20185

--------------------------------------------------------------------------------------------------------------------
-> Cond = 3

             Predicted number of events
-------------------------------------------------------------
     Percentiles      Smallest
 1%     .0190095       .0190095
 5%     .0693943       .0461797
10%     .1261393       .0508793       Obs                70
```

© 2019 Chang JH et al. *JAMA Network Open.*

```
25%     .3660958      .0693943      Sum of Wgt.         70

50%     1.755321                  Mean          33.47019
                    Largest      Std. Dev.      172.9596
75%     10.31682      60.31218
90%     31.80958      183.7684      Variance      29915.01
95%     60.31218      238.2762      Skewness      7.676411
99%     1427.773      1427.773      Kurtosis      62.13217


.
.
.
. nbreg GunTime Condition_Gun Condition_Sword Sex GunQMn BehMean MediaExpS GunInt Age AnyGunsInHouse GunSafety, clu
> ster(Pair)

Fitting Poisson model:

Iteration 0:   log pseudolikelihood = -10997.047
Iteration 1:   log pseudolikelihood = -10994.005
Iteration 2:   log pseudolikelihood = -10994.005

Fitting constant-only model:

Iteration 0:   log pseudolikelihood = -1001.5195
Iteration 1:   log pseudolikelihood = -671.59079
Iteration 2:   log pseudolikelihood = -671.52093
Iteration 3:   log pseudolikelihood = -671.52091

Fitting full model:

Iteration 0:   log pseudolikelihood = -662.84693
Iteration 1:   log pseudolikelihood = -656.98485
Iteration 2:   log pseudolikelihood = -655.60209
```

© 2019 Chang JH et al. *JAMA Network Open.*

Iteration 3: log pseudolikelihood = -655.58901
Iteration 4: log pseudolikelihood = -655.589

Negative binomial regression Number of obs = 205
Wald chi2(10) = 57.62
Dispersion = mean Prob > chi2 = 0.0000
Log pseudolikelihood = -655.589 Pseudo R2 = 0.0237

(Std. Err. adjusted for 108 clusters in Pair)

GunTime	Coef.	Robust Std. Err.	z	P>|z|	[95% Conf. Interval]	
Condition_Gun	.9381426	.6056702	1.55	0.121	-.2489492	2.125234
Condition_Sword	1.096266	.6685329	1.64	0.101	-.2140339	2.406567
Sex	.1163542	.3482742	0.33	0.738	-.5662506	.7989591
GunQMn	-.9139013	.362132	-2.52	0.012	-1.623667	-.2041357
BehMean	1.439592	.4896752	2.94	0.003	.4798466	2.399338
MediaExpS	.0347457	.1076068	0.32	0.747	-.1761598	.2456511
GunInt	.5017217	.153473	3.27	0.001	.2009202	.8025233
Age	-.2967501	.1247082	-2.38	0.017	-.5411737	-.0523265
AnyGunsInHouse	.5678246	.5243892	1.08	0.279	-.4599594	1.595609
GunSafety	-2.25475	.6207003	-3.63	0.000	-3.4713	-1.038199
_cons	6.365413	1.778243	3.58	0.000	2.88012	9.850705
/lnalpha	1.973591	.1435721			1.692195	2.254987
alpha	7.196471	1.033213			5.431387	9.535169

.
. predict GunTime_full_Predicted, n
(15 missing values generated)

© 2019 Chang JH et al. *JAMA Network Open.*

```
.
. by Cond: summarize GunTime_full_Predicted, detail

------------------------------------------------------------------------------------------------------------------------
-> Cond = 1

              Predicted number of events
-------------------------------------------------------------
     Percentiles      Smallest
 1%     .4991218       .4991218
 5%     .7290981       .6229148
10%     2.123777       .6700597       Obs                69
25%     4.686748       .7290981       Sum of Wgt.          69

50%      15.1519                      Mean           25.29234
                    Largest       Std. Dev.      38.77802
75%     21.56497       106.4454
90%     72.38731       109.1912       Variance       1503.735
95%     106.4454       133.4189       Skewness        3.32254
99%      241.966        241.966       Kurtosis       16.44027

------------------------------------------------------------------------------------------------------------------------
-> Cond = 2

              Predicted number of events
-------------------------------------------------------------
     Percentiles      Smallest
 1%     1.153433       1.153433
 5%     2.539562       1.613829
10%     5.113981        2.40022       Obs                66
25%     12.75492       2.539562       Sum of Wgt.          66

50%     33.38746                      Mean           135.7823
                    Largest       Std. Dev.      303.6202
```

© 2019 Chang JH et al. *JAMA Network Open.*

```
75%    88.27286      530.8691
90%    343.7274      1007.138      Variance      92185.24
95%    530.8691      1313.908      Skewness      3.918153
99%    1813.469      1813.469      Kurtosis      19.2524

---------------------------------------------------------------------------------------------------------------
-> Cond = 3

              Predicted number of events
-------------------------------------------------------------
     Percentiles      Smallest
 1%    1.120996      1.120996
 5%    4.314032      2.534232
10%    6.765446      2.538124      Obs                70
25%    19.25331      4.314032      Sum of Wgt.        70

50%    41.43936                    Mean          122.3574
                    Largest        Std. Dev.     308.8151
75%      104.22      512.9313
90%     331.112      513.9496      Variance      95366.75
95%    512.9313      545.1101      Skewness      6.328484
99%    2447.313      2447.313      Kurtosis      47.38929


.
.
.
. nbreg SelfOther Condition_Gun Condition_Sword Sex GunQMn BehMean MediaExpS GunInt Age AnyGunsInHouse GunSafety, c
> luster(Pair)

Fitting Poisson model:

Iteration 0:   log pseudolikelihood =  -518.2505
Iteration 1:   log pseudolikelihood = -513.78261
```

© 2019 Chang JH et al. *JAMA Network Open.*

```
Iteration 2:   log pseudolikelihood =  -513.7114
Iteration 3:   log pseudolikelihood = -513.71138

Fitting constant-only model:

Iteration 0:   log pseudolikelihood = -339.48177
Iteration 1:   log pseudolikelihood = -151.59496
Iteration 2:   log pseudolikelihood = -151.52021
Iteration 3:   log pseudolikelihood = -151.52013
Iteration 4:   log pseudolikelihood = -151.52013

Fitting full model:

Iteration 0:   log pseudolikelihood = -148.16189  (not concave)
Iteration 1:   log pseudolikelihood = -143.02753
Iteration 2:   log pseudolikelihood = -139.04037
Iteration 3:   log pseudolikelihood = -136.67917
Iteration 4:   log pseudolikelihood = -136.54239
Iteration 5:   log pseudolikelihood = -136.54193
Iteration 6:   log pseudolikelihood = -136.54193

Negative binomial regression                    Number of obs     =        205
                                                Wald chi2(10)     =     113.99
Dispersion           = mean                     Prob > chi2       =     0.0000
Log pseudolikelihood = -136.54193               Pseudo R2         =     0.0989

                                    (Std. Err. adjusted for 108 clusters in Pair)
-----------------------------------------------------------------------------
                 |               Robust
      SelfOther  |      Coef.   Std. Err.      z    P>|z|     [95% Conf. Interval]
----------------+------------------------------------------------------------
  Condition_Gun  |   1.929443   .9131453     2.11   0.035      .139711    3.719175
Condition_Sword  |    2.50358   .9500812     2.64   0.008     .6414548    4.365705
            Sex  |  -.2898032   .5640914    -0.51   0.607    -1.395402    .8157958
```

© 2019 Chang JH et al. *JAMA Network Open.*

```
        GunQMn |  -.2107497   .5234226    -0.40   0.687    -1.236639    .8151396
       BehMean |   3.246105   .7484483     4.34   0.000     1.779173    4.713036
     MediaExpS |   .6297028   .1900981     3.31   0.001     .2571174    1.002288
        GunInt |   1.011085   .1956327     5.17   0.000     .6276521    1.394518
           Age |  -.3904776   .1323407    -2.95   0.003    -.6498606   -.1310946
AnyGunsInHouse |  -3.001787   1.036508    -2.90   0.004    -5.033305   -.9702687
     GunSafety |   1.184803    1.10168     1.08   0.282     -.974451    3.344057
         _cons |  -6.133177   2.237096    -2.74   0.006     -10.5178    -1.74855
---------------+----------------------------------------------------------------
      /lnalpha |   2.741236   .2948757                       2.16329    3.319182
---------------+----------------------------------------------------------------
         alpha |   15.50614   4.572383                      8.699714    27.63772
--------------------------------------------------------------------------------

.
. predict SelfOther_full_Predicted, n
(15 missing values generated)

.
. by Cond: summarize SelfOther_full_Predicted, detail

-------------------------------------------------------------------------------------------------------------------
-> Cond = 1

                 Predicted number of events
-------------------------------------------------------------
      Percentiles      Smallest
 1%     7.90e-06       7.90e-06
 5%     .0001116        .000051
10%     .0003569       .0000746       Obs                  69
25%     .0042411       .0001116       Sum of Wgt.          69

50%     .0290138                      Mean           2.508161
                        Largest       Std. Dev.      13.00765
```

© 2019 Chang JH et al. *JAMA Network Open.*

```
75%      .103546      .6756673
90%     .4075507       17.7336      Variance      169.1989
95%     .6756673      59.63063      Skewness      5.751722
99%     89.81816      89.81816      Kurtosis      35.86833

---------------------------------------------------------------------------------------------------------------
-> Cond = 2

                Predicted number of events
-------------------------------------------------------------
      Percentiles      Smallest
 1%    .0006997       .0006997
 5%     .005785       .001184
10%     .0167463      .0039142      Obs               66
25%     .0790556      .005785       Sum of Wgt.       66

50%     .4702139                    Mean          87.00368
                      Largest       Std. Dev.     529.6397
75%     2.422721      154.4388
90%     21.97327      292.5996      Variance      280518.2
95%     154.4388      878.9033      Skewness      7.439806
99%     4224.587      4224.587      Kurtosis      58.26019

---------------------------------------------------------------------------------------------------------------
-> Cond = 3

                Predicted number of events
-------------------------------------------------------------
      Percentiles      Smallest
 1%     .001309       .001309
 5%      .00724       .0032514
10%     .0088509      .0033476      Obs               70
25%     .0393573       .00724       Sum of Wgt.       70
```

© 2019 Chang JH et al. *JAMA Network Open.*

50%	.2314704		Mean	13.4211
		Largest	Std. Dev.	52.22407
75%	2.522074	34.00886		
90%	19.01313	168.8974	Variance	2727.354
95%	34.00886	268.6051	Skewness	4.775398
99%	310.9273	310.9273	Kurtosis	25.02488

© 2019 Chang JH et al. *JAMA Network Open.*
