# Supplementary material for: Effect of Exposure to Gun Violence in Video Games on Children’s Dangerous Behavior With Real Guns: A Randomized Clinical Trial
Source: JAMA Netw Open. 2019 May 31;2(5):e194319. doi: 10.1001/jamanetworkopen.2019.4319 (PMC6547242; doi:10.1001/jamanetworkopen.2019.4319)
Supplement: Supplement 3. — Data Sharing Statement [file jamanetwopen-2-e194319-s003.pdf]

Data Sharing Statement

Chang. Effect of exposure to gun violence in video games on children's dangerous behavior with real guns: a randomized clinical trial. *JAMA Netw Open*. Published May 31, 2019. 10.1001/jamanetworkopen.2019.4319

Data
Data available: Yes
Data types: Deidentified participant data
How to access data: Data will be held on Dataverse. Please contact the research team for access.
When available: With publication

Supporting Documents
Document types: Statistical/analytic code, Informed consent form
How to access documents: Data will be uploaded to figshare and Dataverse. Please contact the research team for access.
When available: With publication

Additional Information
Who can access the data: Anyone requesting the data
Types of analyses: For any purpose
Mechanisms of data availability: Without investigator support
Any additional restrictions: N/A
